# Supplementary material for: Tuning lithium-peroxide formation and decomposition routes with single-atom catalysts for lithium–oxygen batteries
Source: Nat Commun. 2020 May 4;11:2191. doi: 10.1038/s41467-020-15712-z (PMC7198606; doi:10.1038/s41467-020-15712-z)
Supplement: Supplementary file 1 — Supplementary Information [file 41467_2020_15712_MOESM1_ESM.pdf]

Supplementary Information

Tuning Lithium-Peroxide Formation and Decomposition Routes with Single-Atom Catalysts for Lithium-Oxygen Batteries

By song et al.

Supplementary Figures

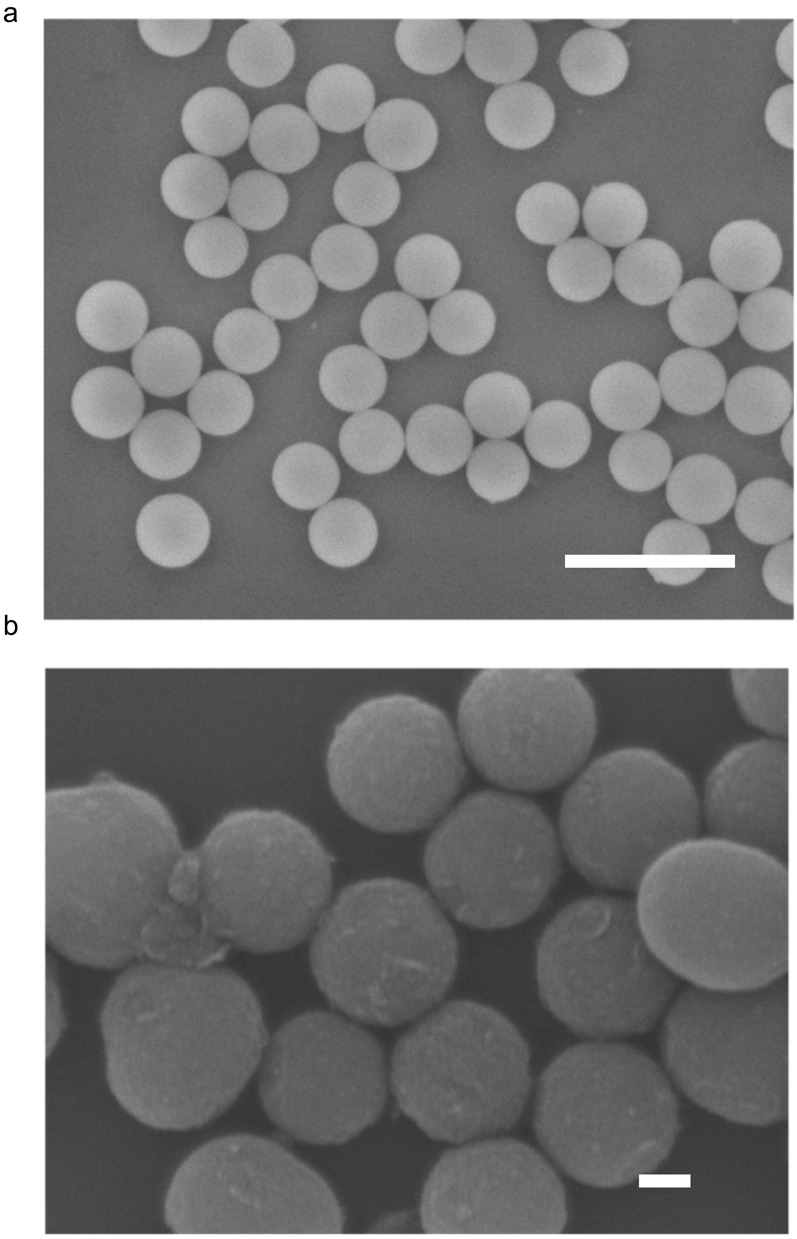

Supplementary Figure 1 | SEM images of the precursors. **a.** SEM image of silica sphere prepared by the Stöber method. **b.** SEM image of SiO_2@PDA@Co(acac)$_2$. Scale bars, 1 µm in **a**, 100 nm in **b**.

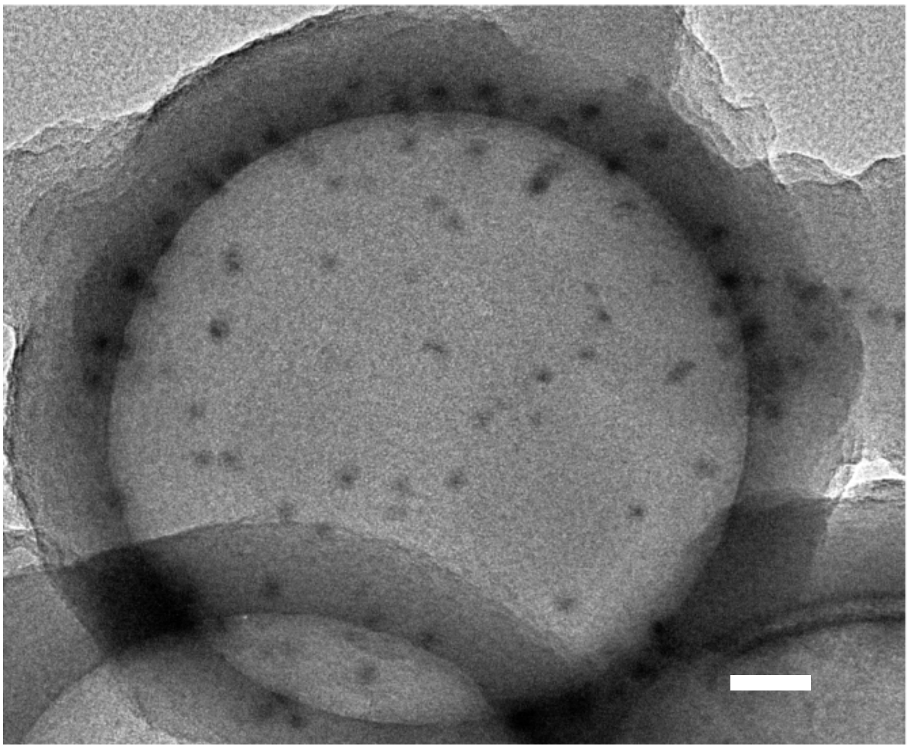

Supplementary Figure 2 | TEM image of N-HP-Co NPs. Scale bar, 50 nm.

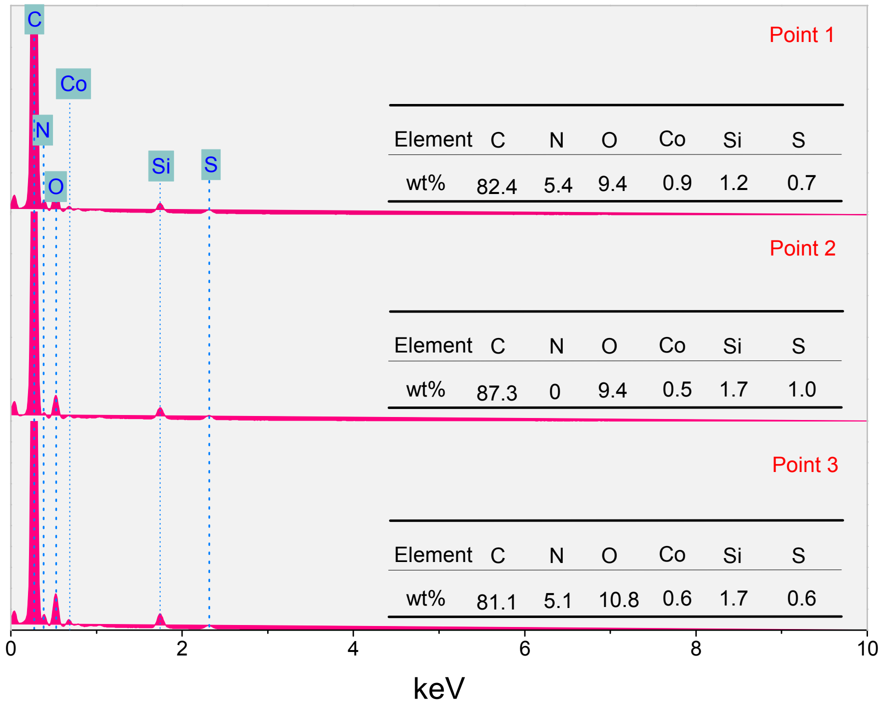

Supplementary Figure 3 | The EDX spectra of N-HP-Co SACs. The result of quantitative analysis of the elements is adopted for references.

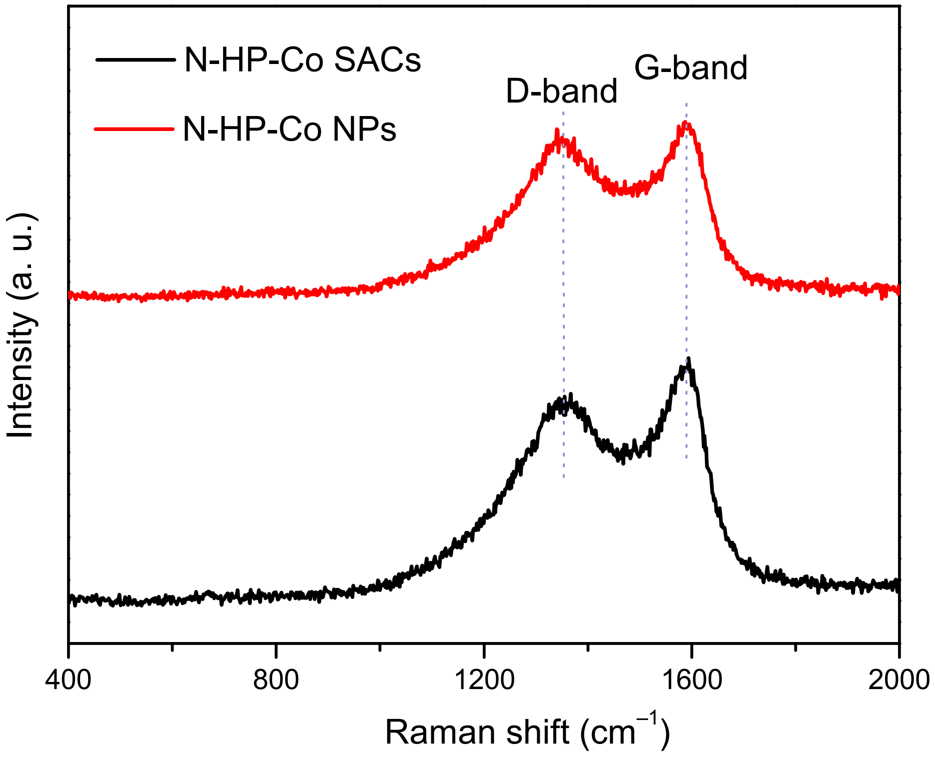

Supplementary Figure 4 | Raman spectra of N-HP-Co NPs and Co SACs. The ratios of I_D and I_G are 0.85 for N-HP-Co SACs and 0.91 for N-HP-Co SAC catalysts.

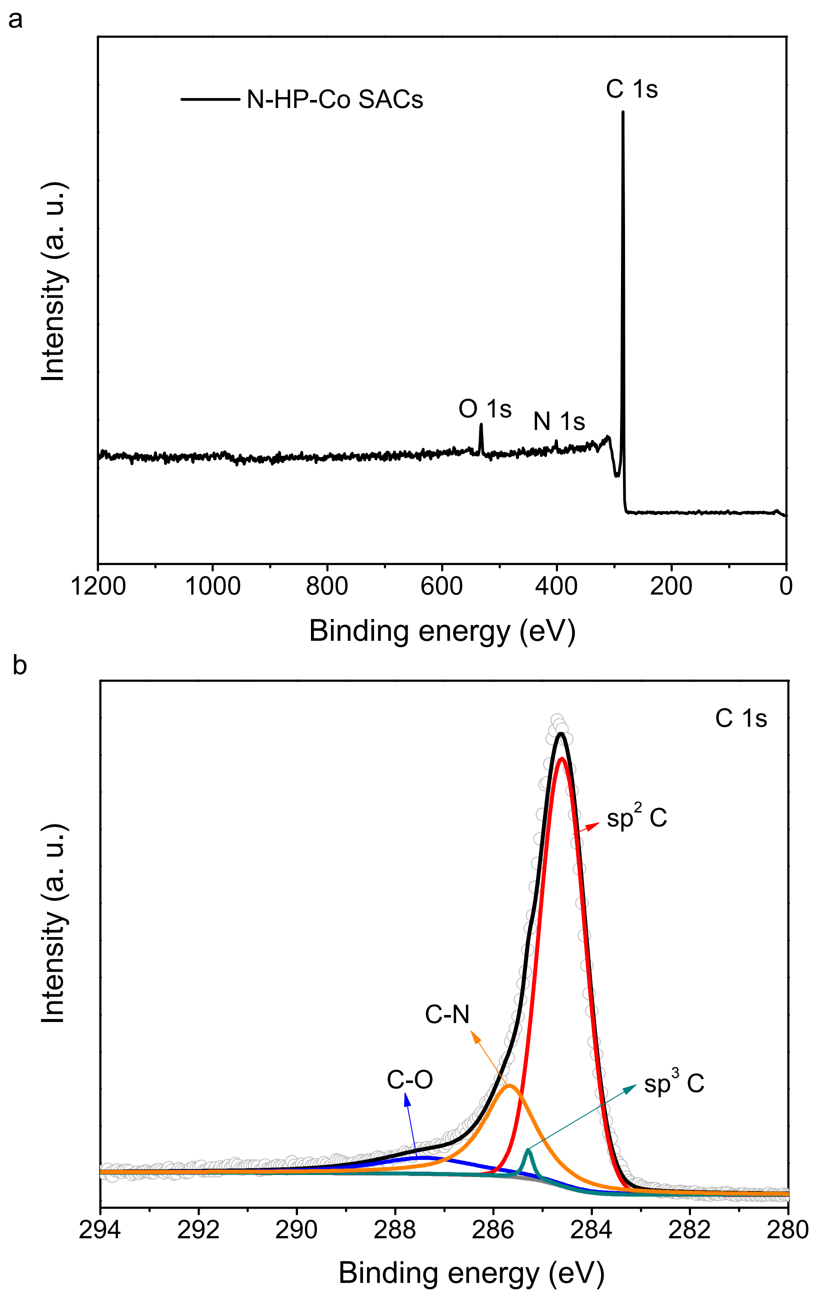

Supplementary Figure 5 | XPS survey and C1s spectra of N-HP-Co SACs.

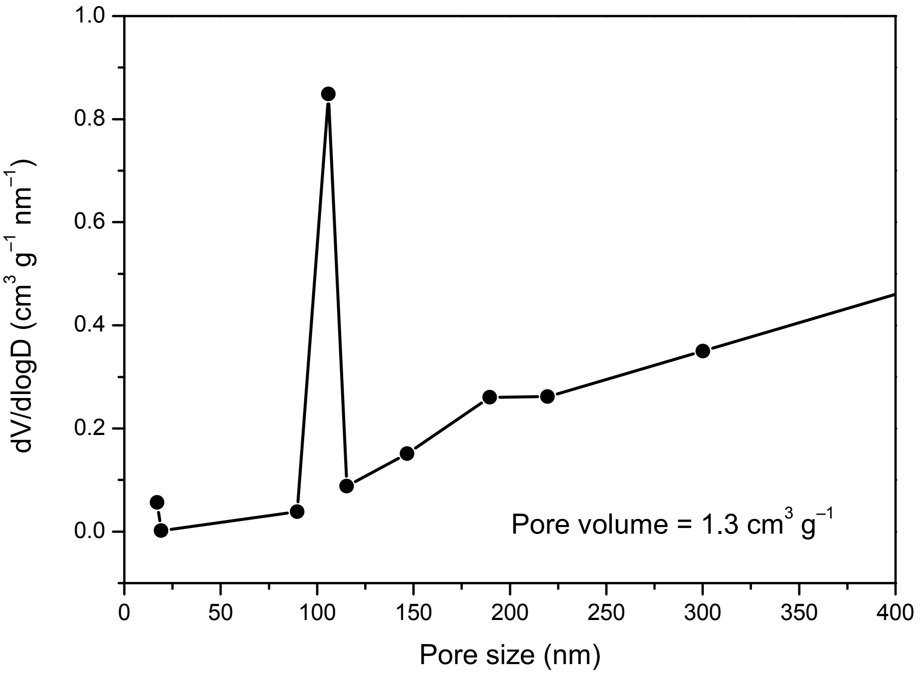

Supplementary Figure 6 | The pore-size distribution of N-HP-Co SACs. The pore volume is 1.3 $cm^3\ g^{-1}$.

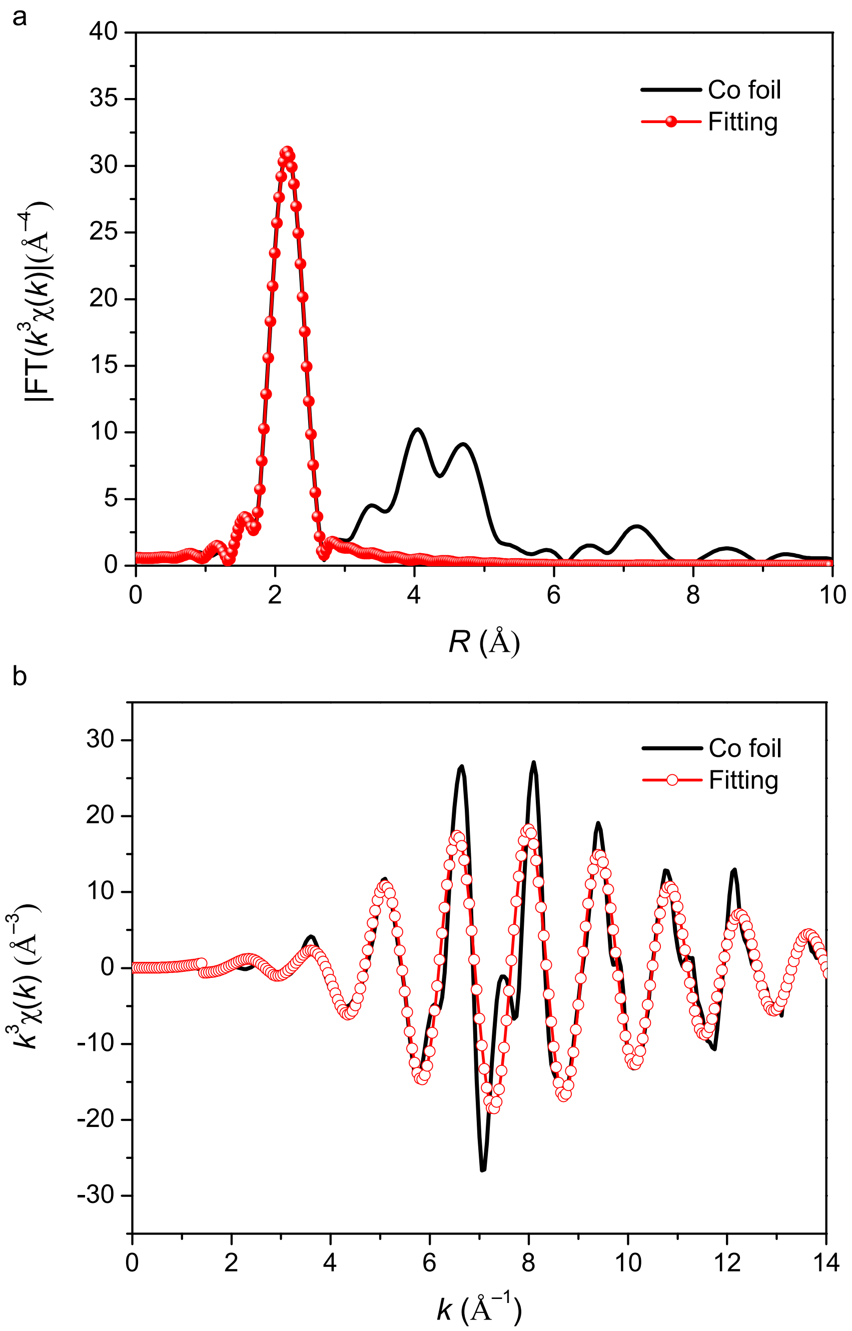

Supplementary Figure 7 | The corresponding EXAFS fitting curves of Co foil. a. *R* space, **b.** *k* space.

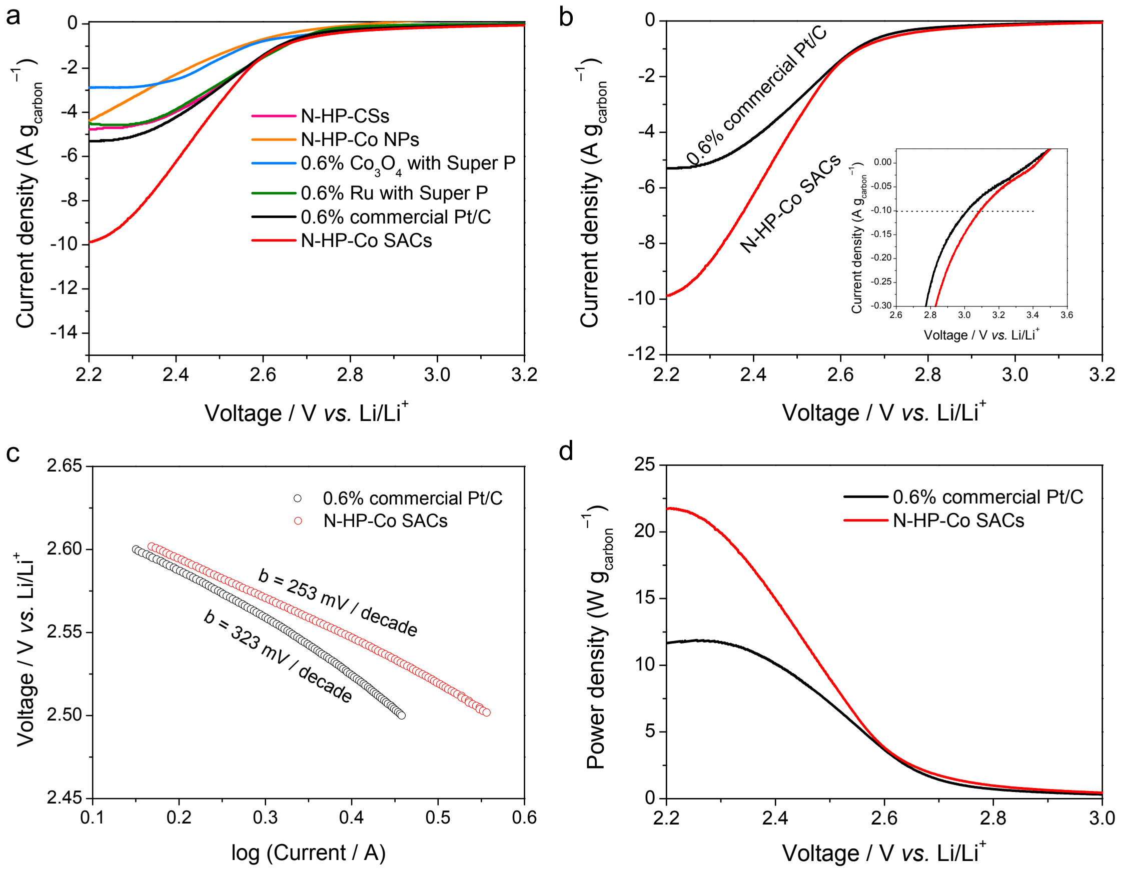

Supplementary Figure 8 | ORR characterization of the common catalysts applied in Li-O_2 batteries. **a.** ORR polarization curves of N-HP-CSs, N-HP-Co NPs, 0.6% Co_3O_4 with Super P, 0.6% Ru with Super P, 0.6% commercial Pt/C and N-HP-Co SACs catalysts in O_2-saturated 1 M LiTFSI in TEGDME with a scan rate of 5 mV s^{-1} at a rotation rate of 900 rpm. **b.** Enlarged ORR polarization of 0.6% commercial Pt/C and N-HP-Co SACs (inset: the current range from − 0.05 to − 0.3 A $g^{-1}{}_{carbon}$). **c, d.** Tafel plot and power density curves of 0.6% commercial Pt/C and N-HP-Co SACs based on the Supplementary Fig. 8b.

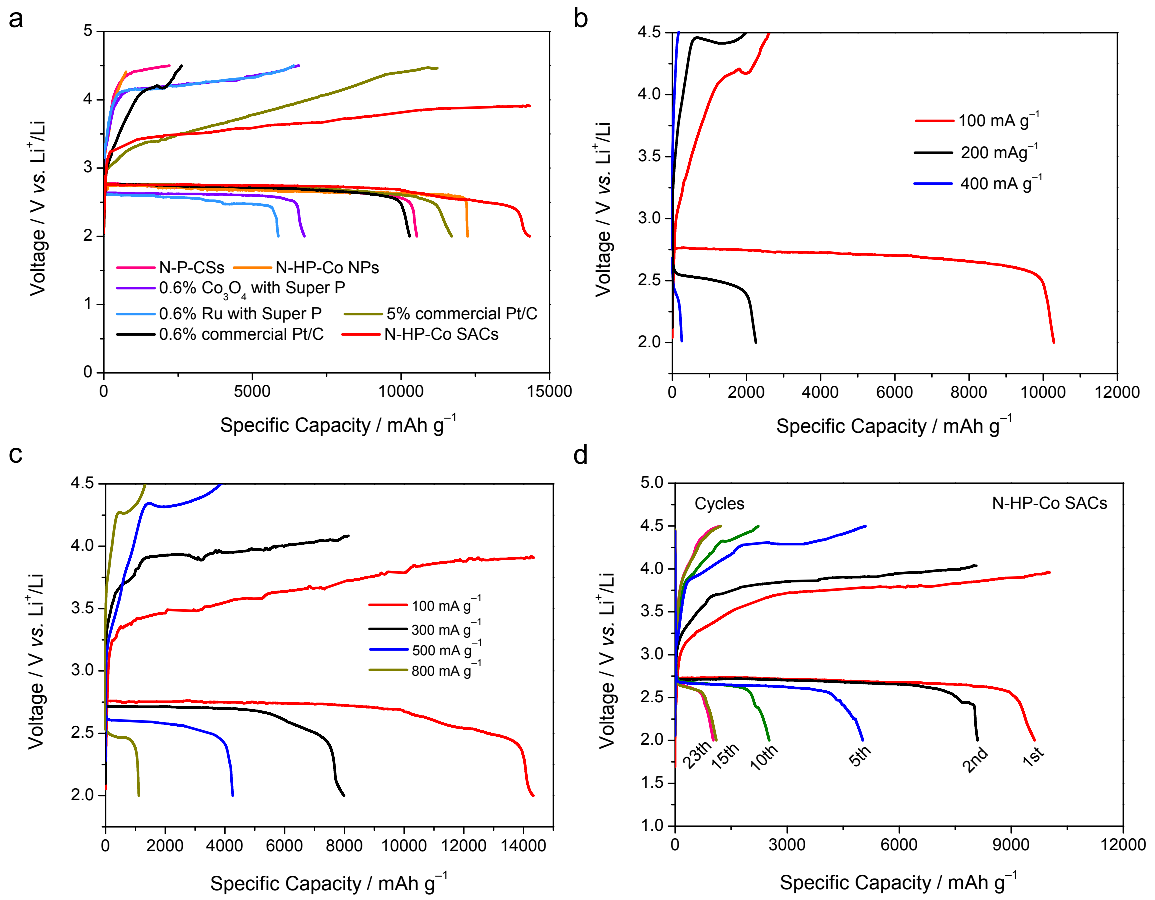

Supplementary Figure 9 | The electrochemistry performance. a. Full range tests of the Li-O_2 cells with the cathodes loaded with 0.6% commercial Pt/C, the common catalysts used in Li-O_2 batteries and N-HP-Co SACs at a current density of 100 mA g^{-1}. The cathodes were discharged with the cut off voltage limited to 2 V. **b.** The rate capability of the Li-O_2 batteries with 0.6% commercial Pt/C at a current densities of 100 mA g^{-1}, 200 mA g^{-1} and 400 mA g^{-1}, respectively. **c.** The rate capability of the Li-O_2 batteries with the N-HP-Co SACs at current densities of 100 mA g^{-1}, 300 mA g^{-1}, 500 mA g^{-1} and 800 mA g^{-1}, respectively. **d.** Cycles of full range test of the Li–O_2 cells with N-HP-Co SACs at a current density of 100 mAh g^{-1}.

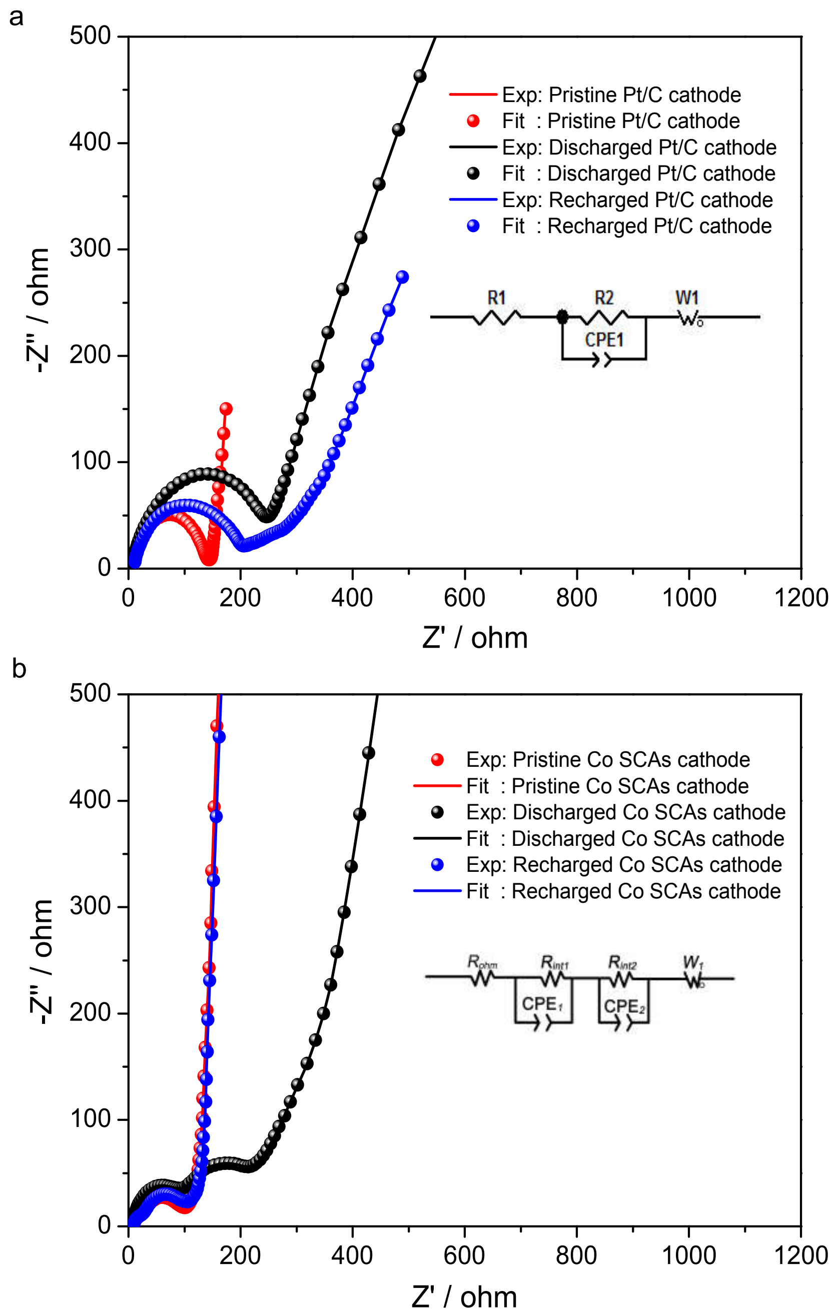

Supplementary Figure 10 | The electrochemical impedance spectra of Li–O_2 cells. a. 0.6% commercial Pt/C catalyst. **b.** N-HP-Co SACs cathodes at different discharge/charge stages.

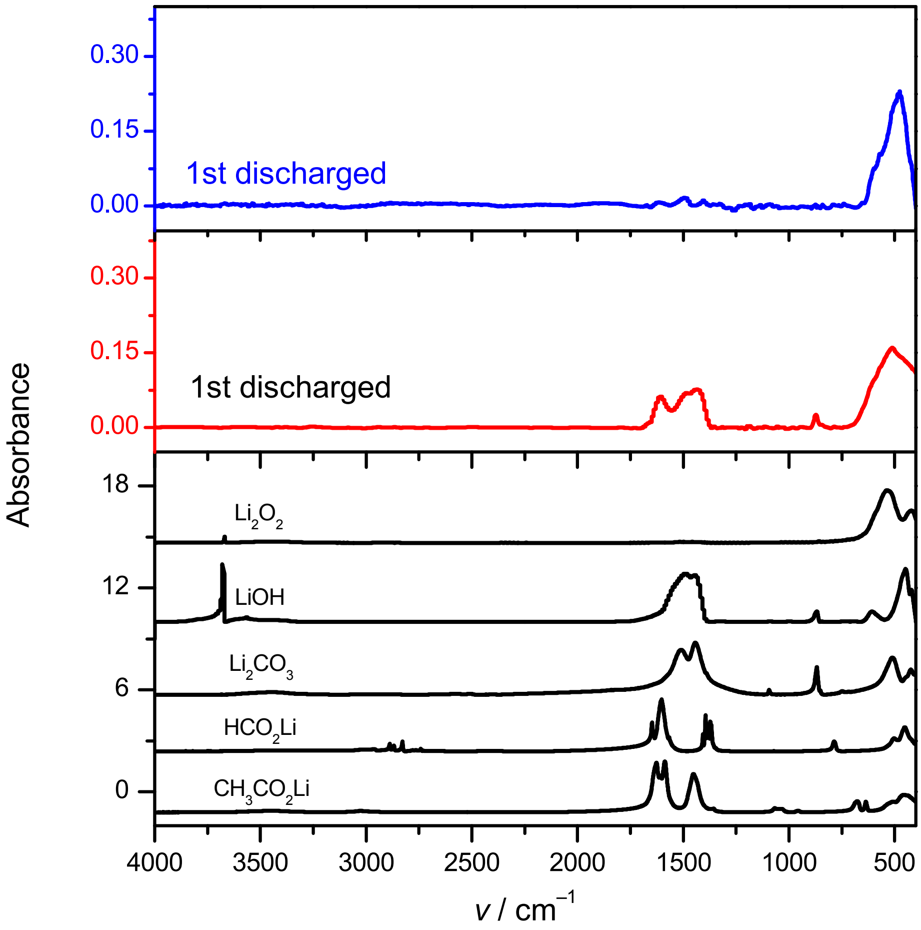

Supplementary Figure 11 | FTIR spectra of the discharged cathodes. 0.6% Commercial Pt/C (black) and N-HP-Co SACs (red) catalysts loaded onto CP cathode after the first discharge at a current density of 100 mA g^{-1} with a limited specific capacity of 3000 mAh g^{-1}. The spectra for Li_2O_2, LiOH, Li_2CO_3, HCO_2Li and CH_3CO_2Li are also shown for reference.

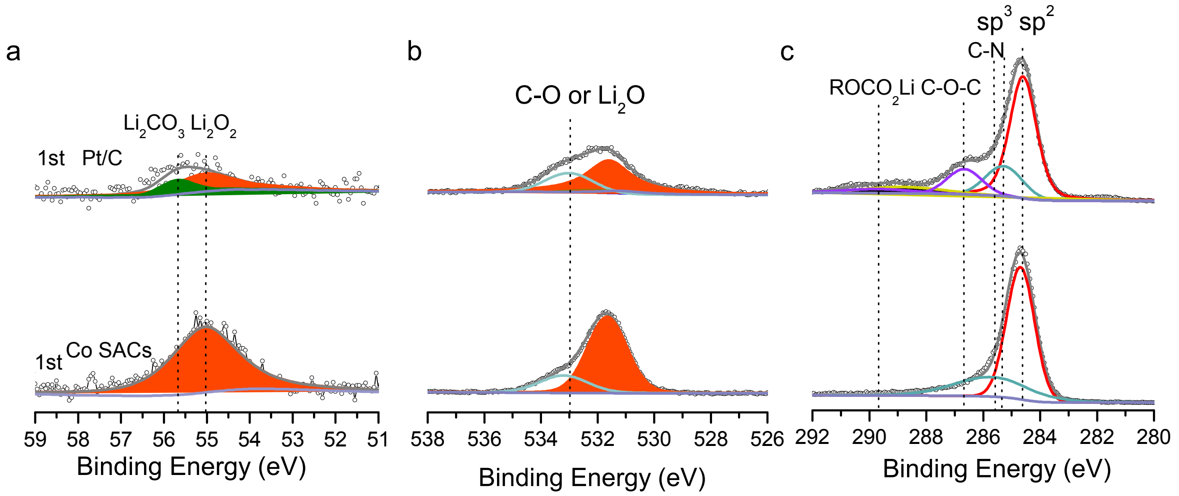

Supplementary Figure 12 | XPS spectra of the discharged cathodes. a. Li 1s, **b.** O 1s, **c.** C 1s after the first discharge at a current density of 100 mA g^{-1} with a limited specific capacity of 3,000 mAh g^{-1}.

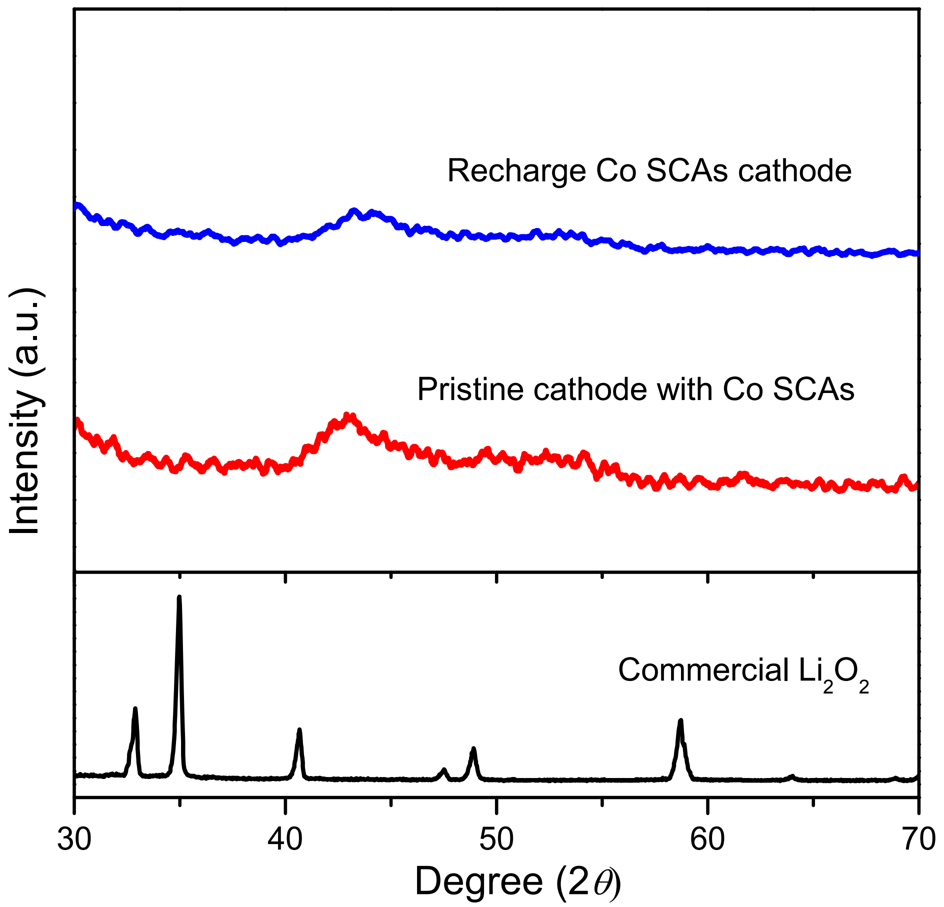

Supplementary Figure 13 | PXRD patterns of pristine and recharging products. a. the 0.6% commercial Pt/C, **b.** N-HP-Co SACs cathode after the first cycle at a current density of 100 mA g^{-1} with a limited specific capacity of 3,000 mAh g^{-1}.

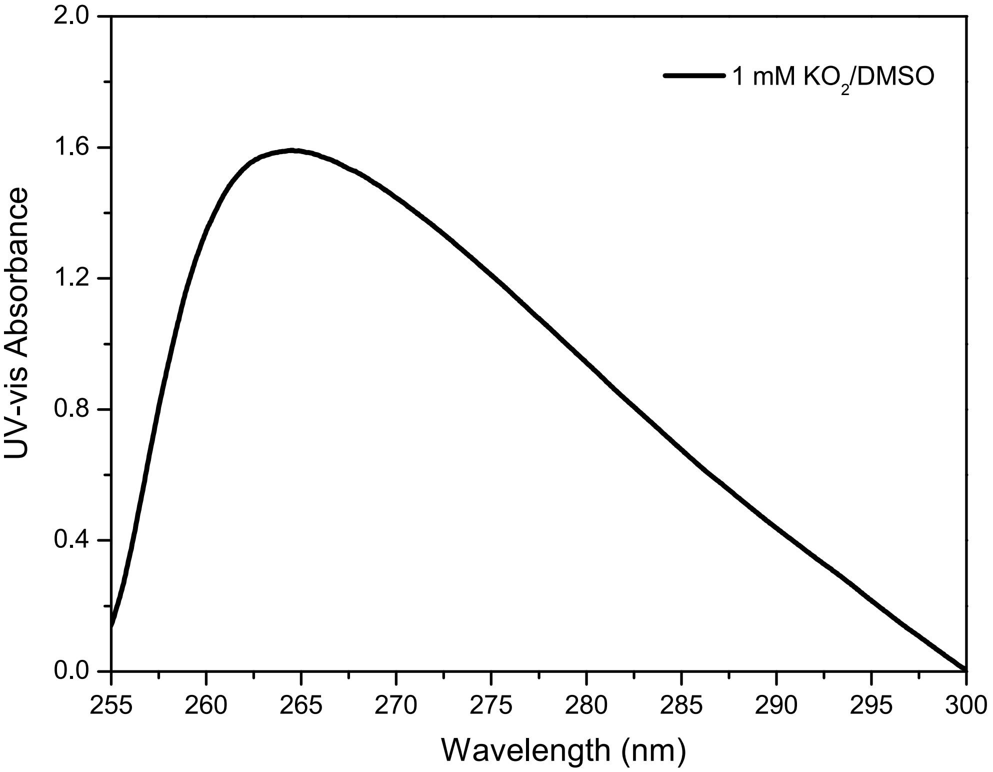

Supplementary Figure 14 | UV-vis spectra. The commercial KO_2 dissolved in DMSO solvent with a superoxide concentration of 1 mM.

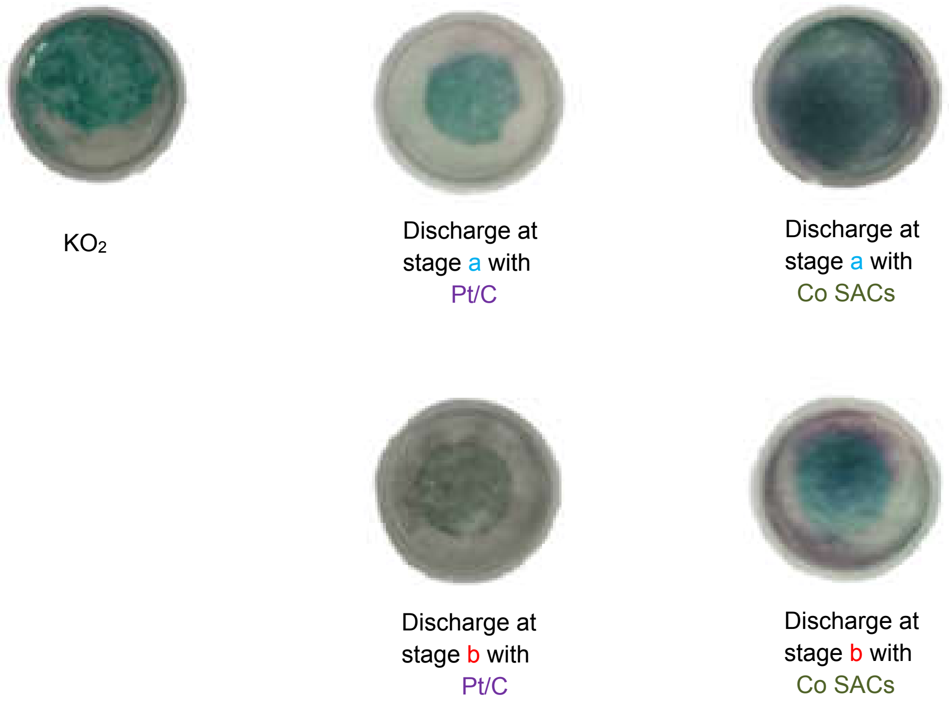

Supplementary Figure 15 | Superoxide detection with nitrotetrazolium blue chloride.

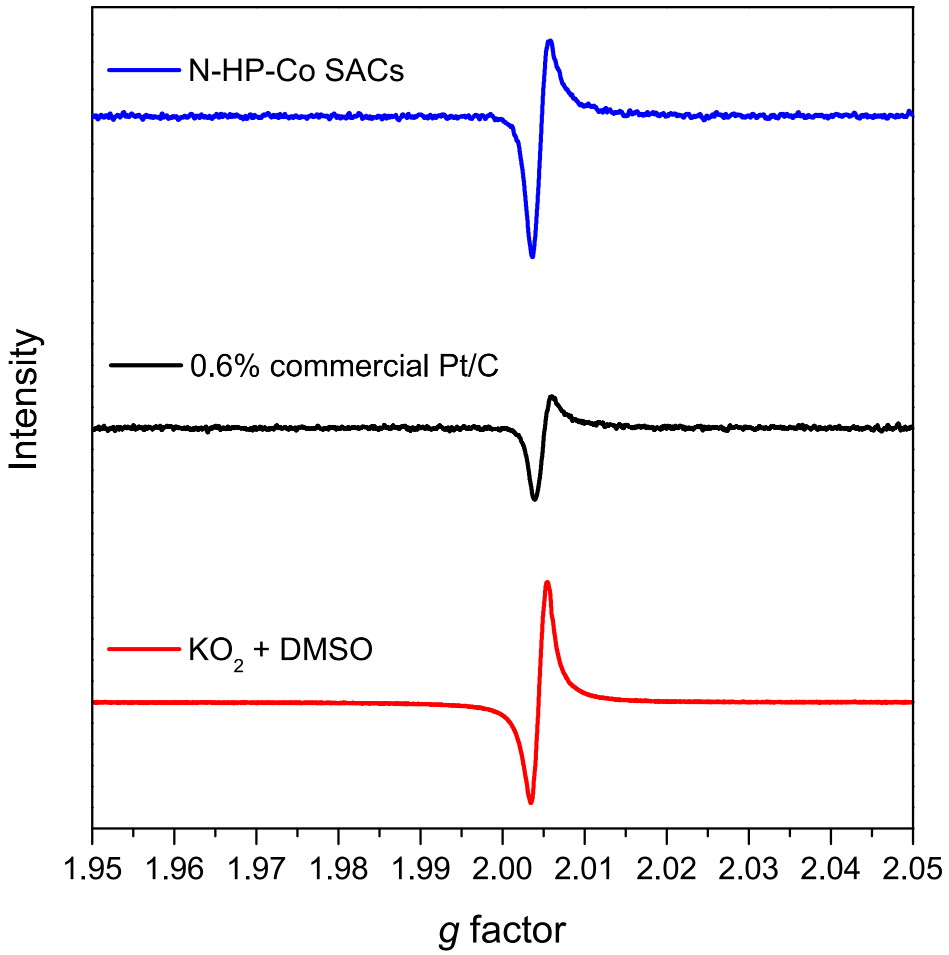

Supplementary Figure 16 | The EPR spectra. The spectra were the KO_2 + DMSO solution and the electrolyte of Li-O_2 batteries with 0.6% commercial Pt/C and N-HP-Co SACs cathodes at half-charged state, respectively.

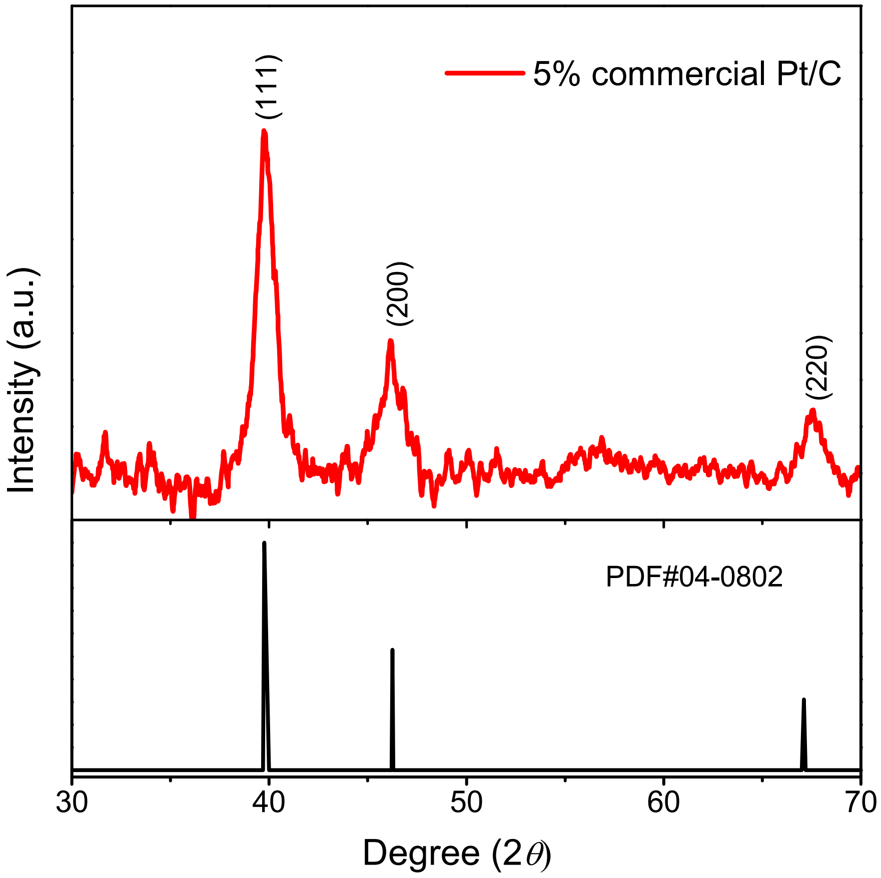

Supplementary Figure 17 | The XRD patterns of the 5% commercial Pt/C.

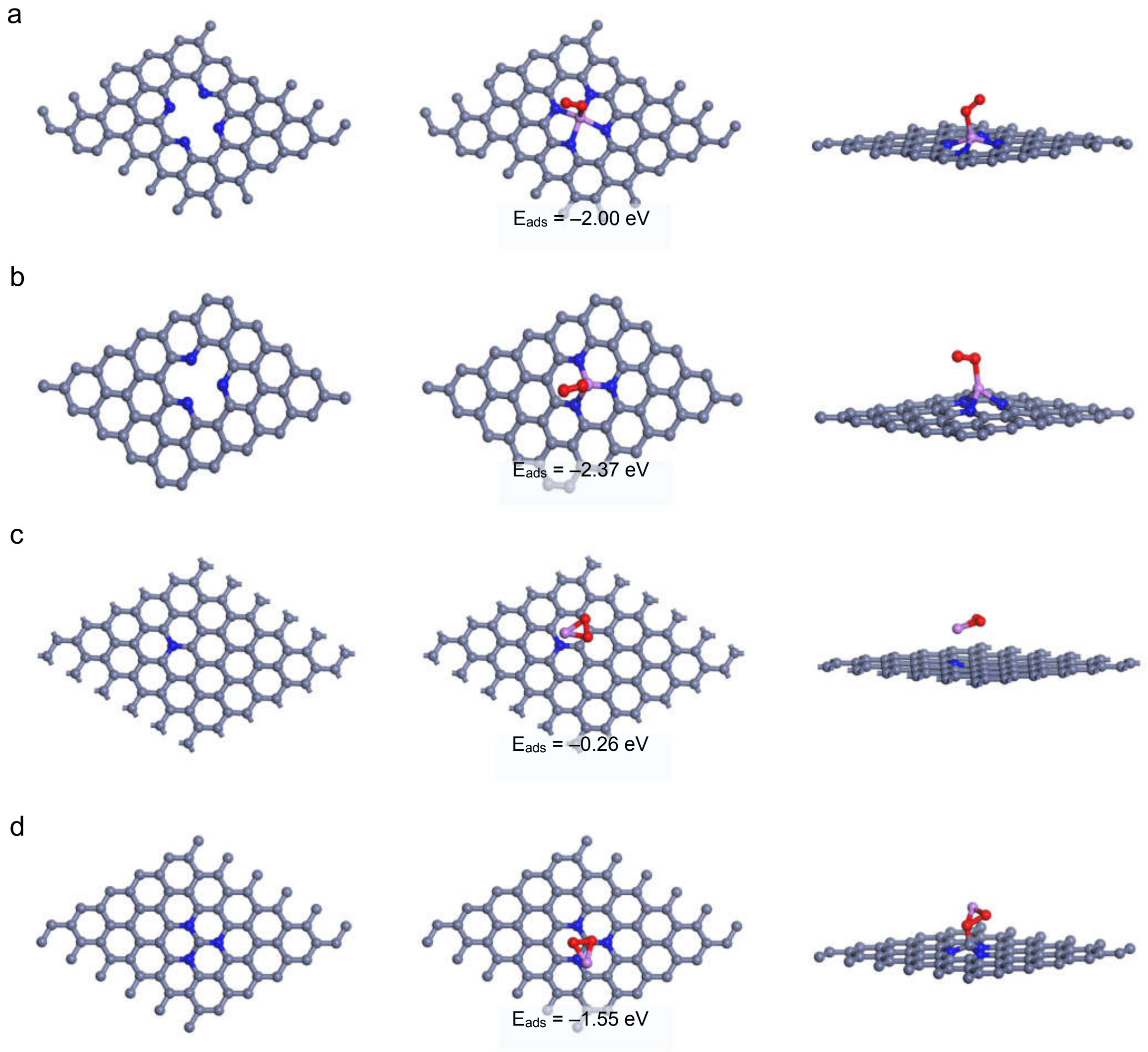

Supplementary Figure 18 | Schematic view of the LiO_2 intermediate on the different types of N-doped carbon support. a. one-defect carbon, **b.** two-defect carbon, **c.** one Nitrogen-doped carbon, **d.** three Nitrogen-doped carbon.

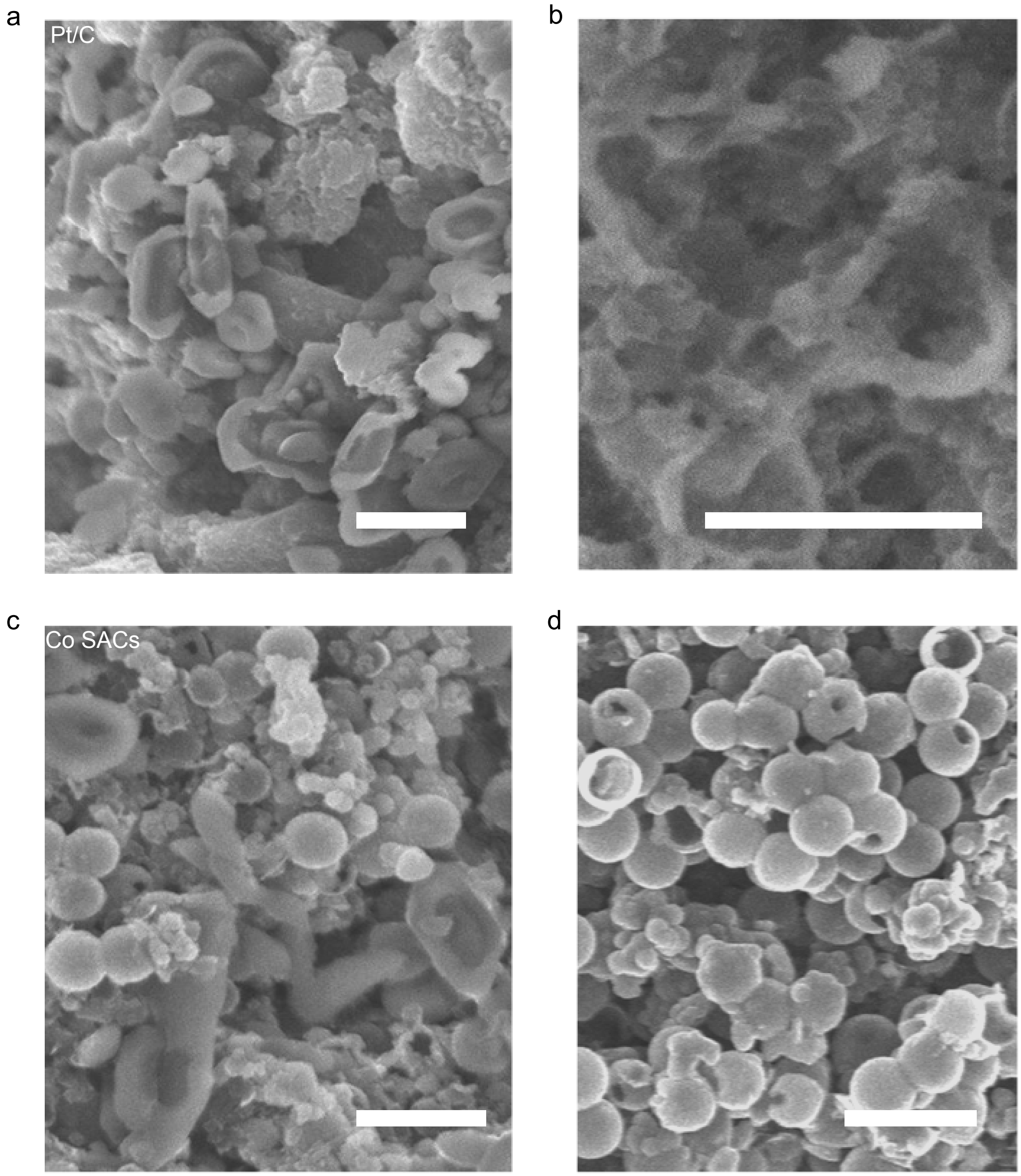

Supplementary Figure 19 | SEM images. Pristine cathodes with commercial Li_2O_2 and recharged cathodes with 0.6% commercial Pt/C (**a**, **b**) and N-HP-Co SACs (**c**, **d**). Scale bars, 1 μm.

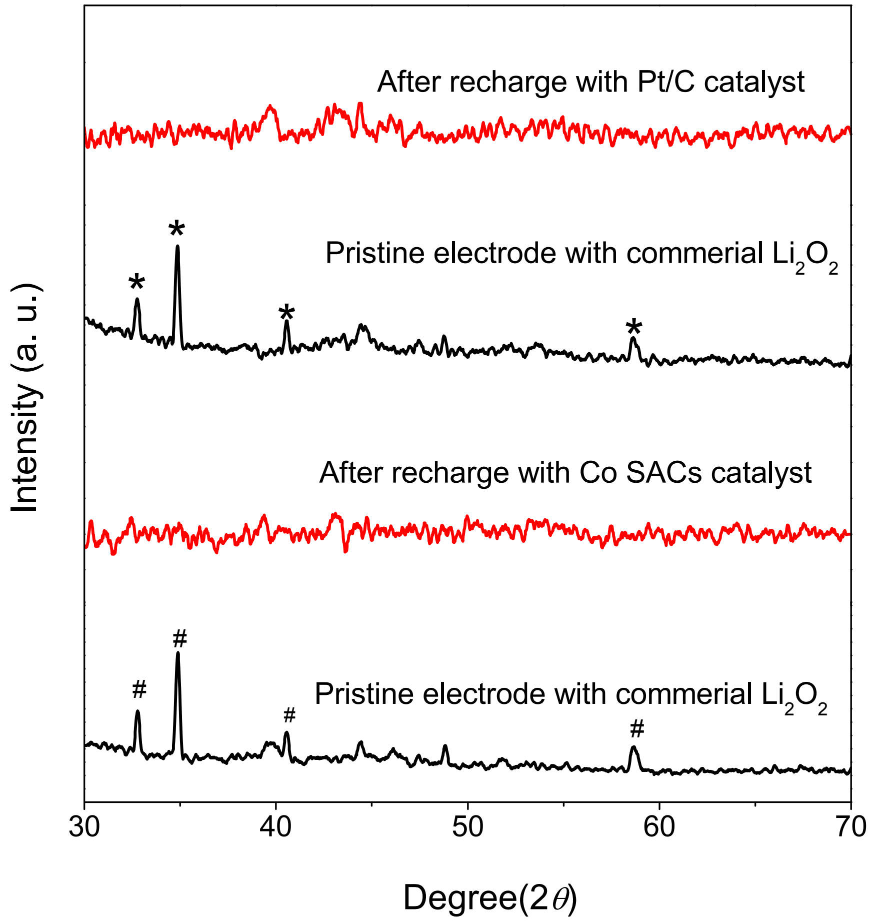

Supplementary Figure 20 | The XRD patterns of different cathodes.

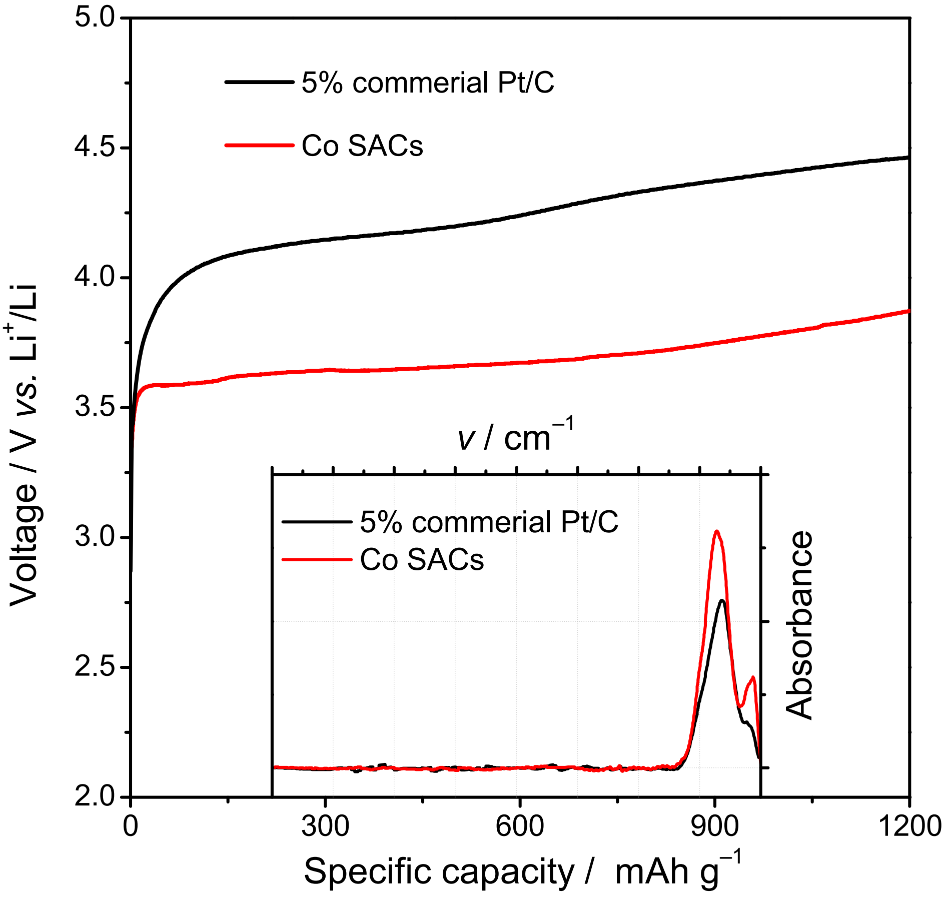

Supplementary Figure 21 | Charge potential profiles of the Li−O_2 cells with different catalysts. The inset is related with the FTIR spectra of the cathodes with different catalysts preloading with commercial Li_2O_2. The current density is 200 mA g^{-1}.

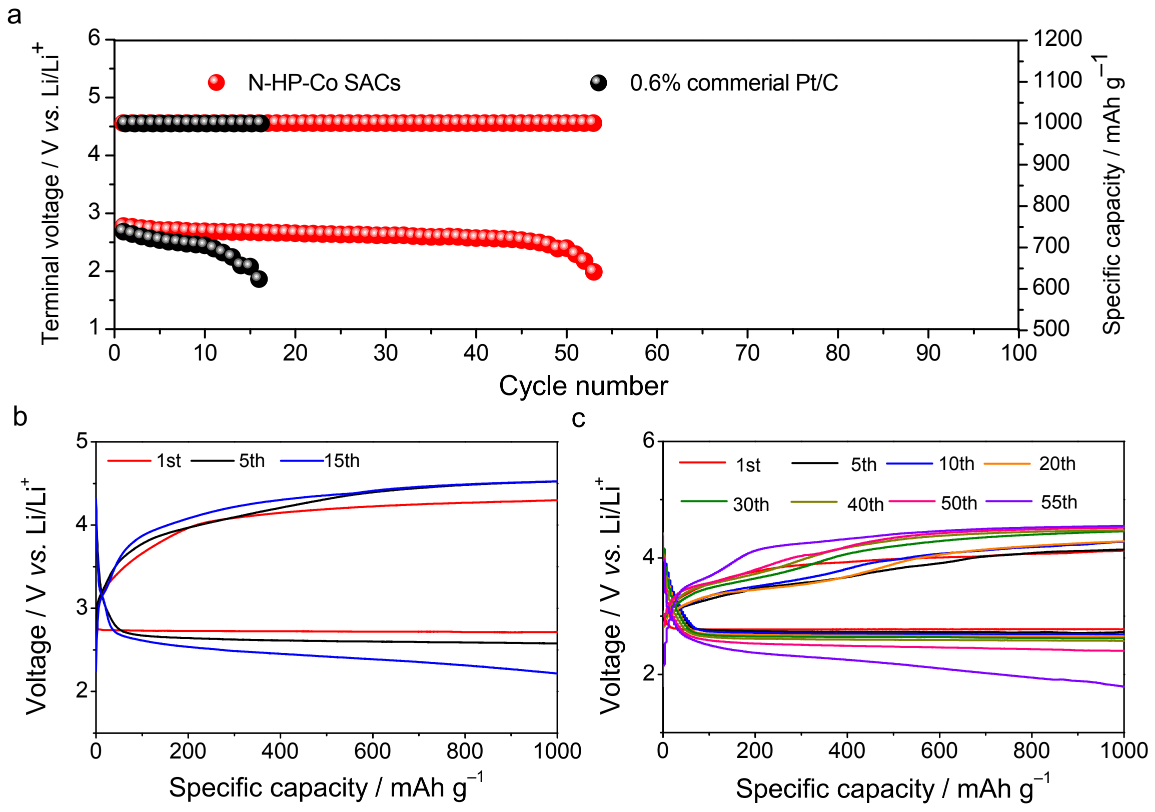

Supplementary Figure 22 | Charge-discharge curves of the catalysts. a. Variation of voltage on the terminal discharge of the Li-O_2 cells with the 0.6% commercial Pt/C catalysts and N-HP-Co SACs loaded onto CP cathodes at a density of 400 mA g^{-1} with a limited specific capacity of 1,000 mAh g^{-1}. **b.** Discharge-charge curves of the Li-O_2 cells the 0.6% commercial Pt/C catalysts and N-HP-Co SACs loaded onto CP cathodes at different cycles.

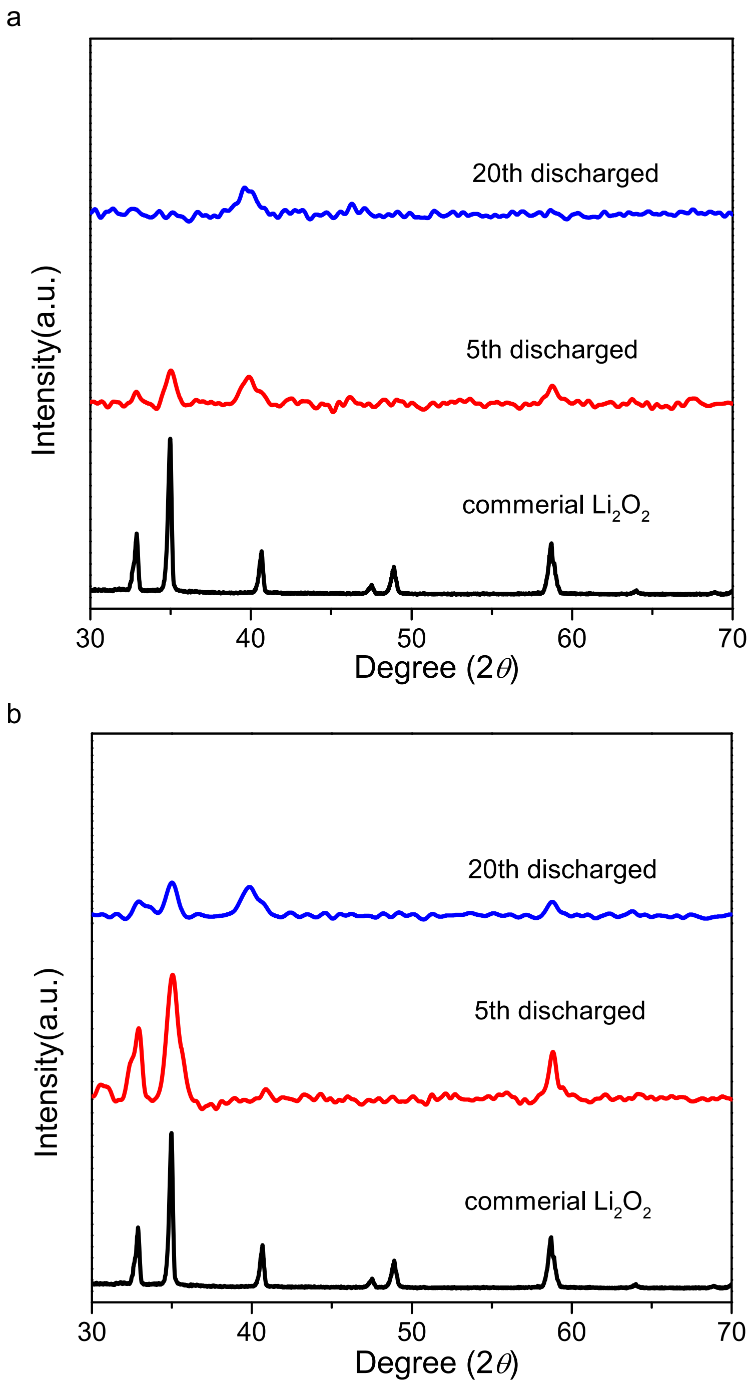

Supplementary Figure 23 | The XRD patterns of the cathodes. a. 0.6% commercial Pt/C. **b.** N-HP-Co SACs catalysts after 5th and 20th cycles. The spectra of Li_2O_2 are also shown for reference.

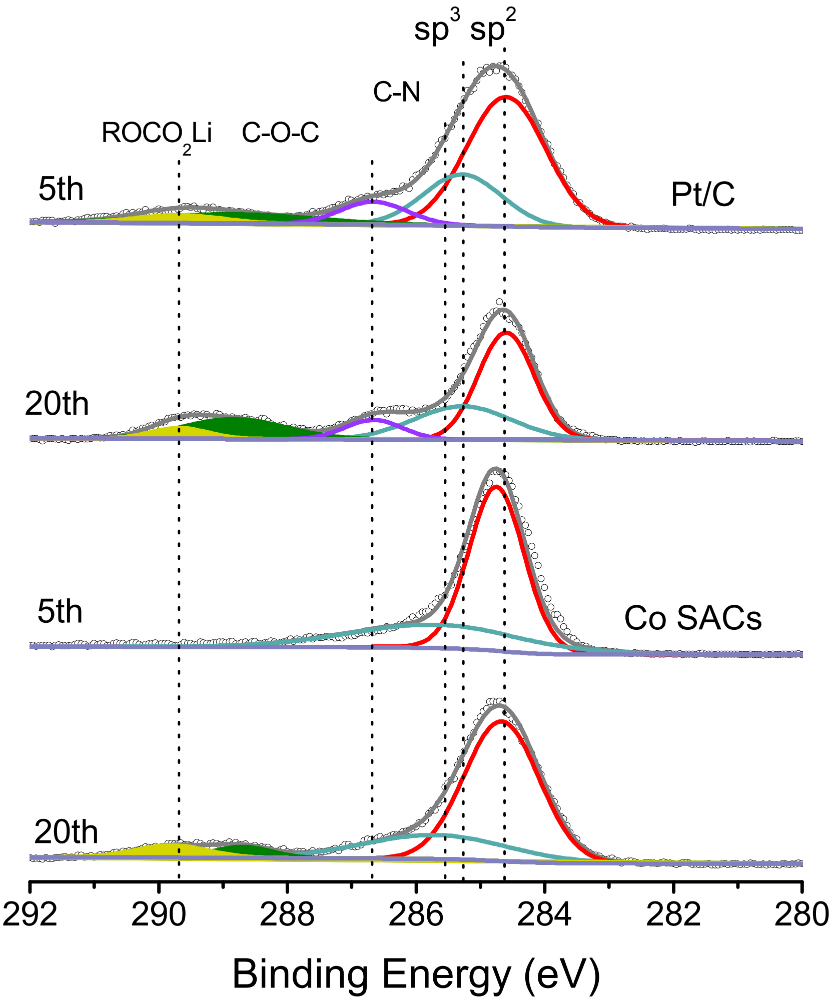

Supplementary Figure 24 | XPS spectra of the discharged cathodes.

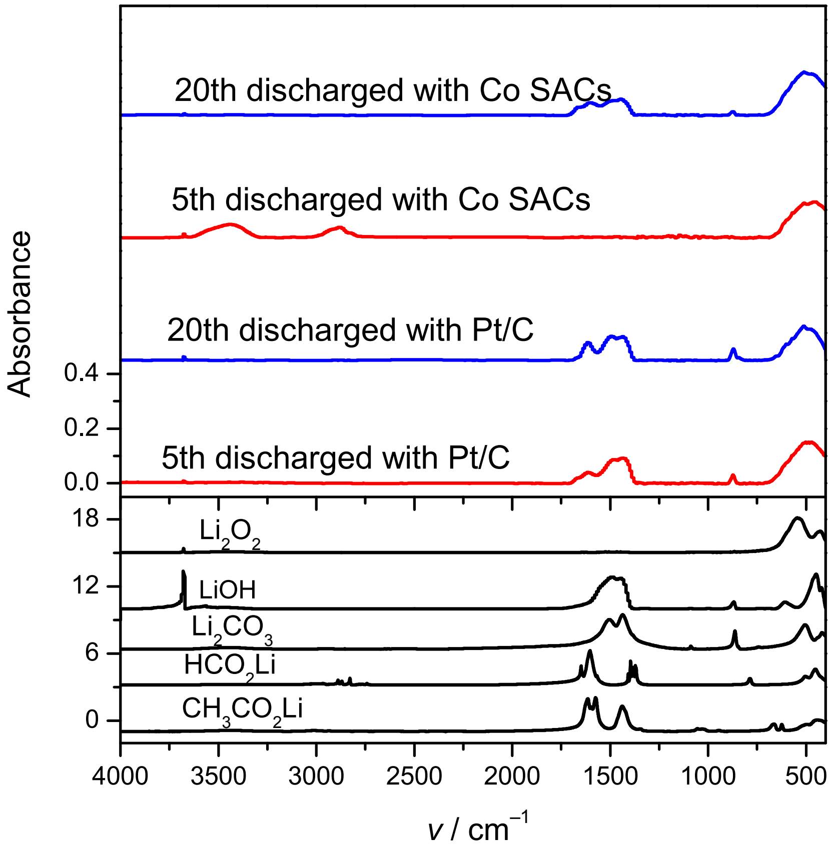

Supplementary Figure 25 | FTIR spectra of the discharge cathodes. Commercial Pt/C and N-HP-Co SACs catalysts loaded onto CP cathodes after different discharge-charge cycles. The spectra for Li_2O_2, LiOH, Li_2CO_3, HCO_2Li and CH_3CO_2Li are also shown for reference.

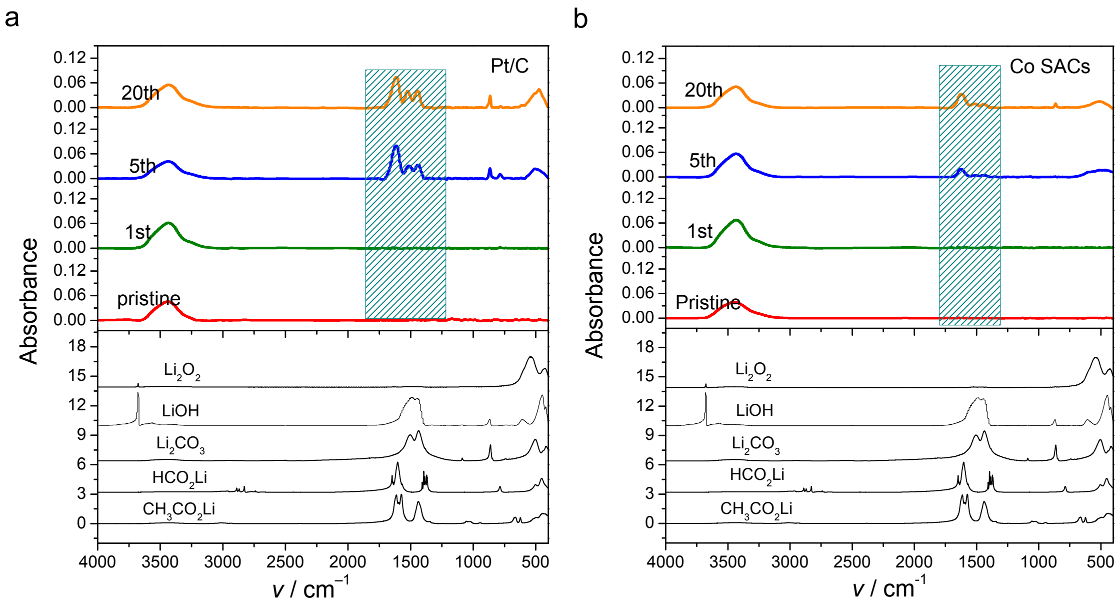

Supplementary Figure 26 | FTIR spectra of the recharged cathodes. a. 0.6% commercial Pt/C after 1st, 5th and 20th recharged process. **b.** N-HP-Co SACs after 1st, 5th and 20th recharged process. The spectra for Li_2O_2, LiOH, Li_2CO_3, HCO_2Li, and CH_3CO_2Li are also shown for reference.

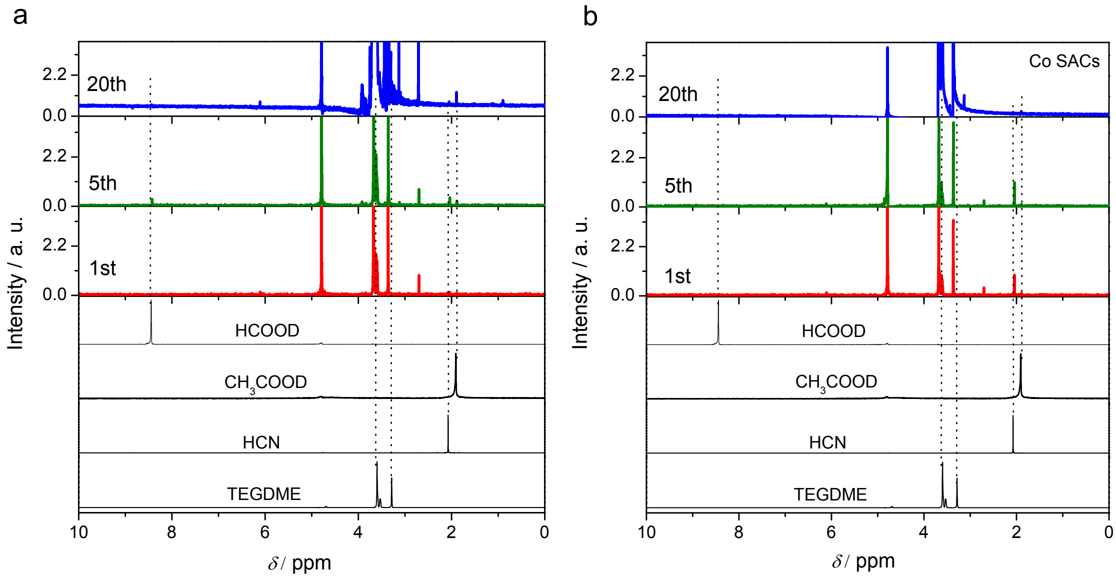

Supplementary Figure 27 | ^{1}H NMR spectra of the recharged cathodes. a. 0.6% commercial Pt/C, **b.** N-HP-Co SACs after 1st, 5th and 20th recharged. The spectra for TEGDME (tetraethylene glycol dimethyl ether), HCN, HCO_2Li and CH_3CO_2Li are also shown for reference.

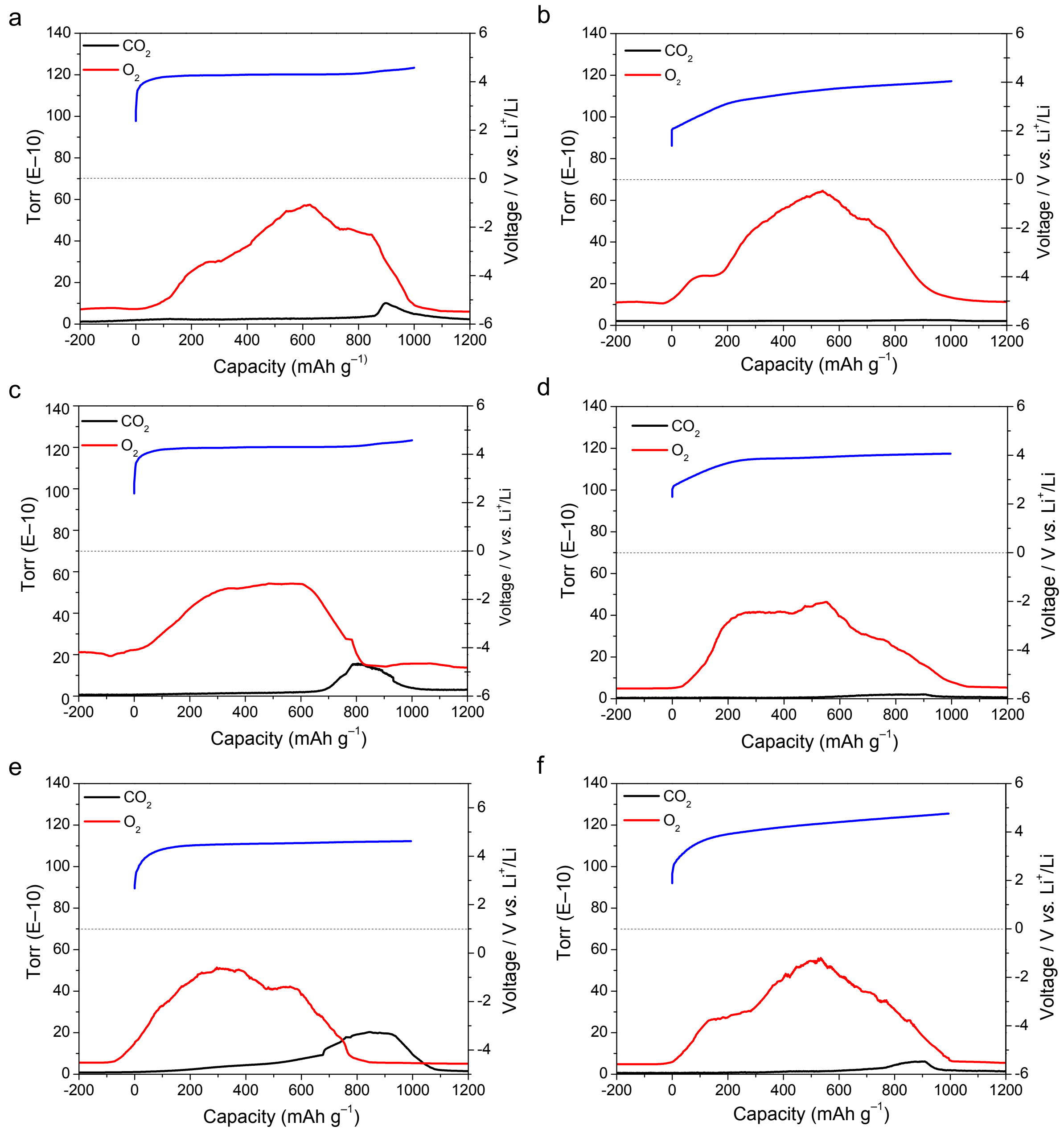

Supplementary Figure 28 | DEMS curve of the Li-O_2 battery charge with the cycles. a, b. DEMS curves of 0.6% commercial Pt/C and the N-HP-Co SACs-based Li-O_2 battery charge with a current of 200 mA g^{-1} and a specific capacity of 1, 000 mAh g^{-1} after the 1st recharge. **c**, **d.** DEMS curves of 0.6% commercial Pt/C and the N-HP-Co SACs-based Li-O_2 battery charge with a current of 200 mA g^{-1} and a specific capacity of 1, 000 mAh g^{-1} after the 5th recharge. **e**, **f.** DEMS curves of 0.6% commercial Pt/C and the N-HP-Co SACs-based Li-O_2 battery charge with a current of 200 mA g^{-1} and a specific capacity of 1, 000 mAh g^{-1} after 20th and 50th recharge, respectively.

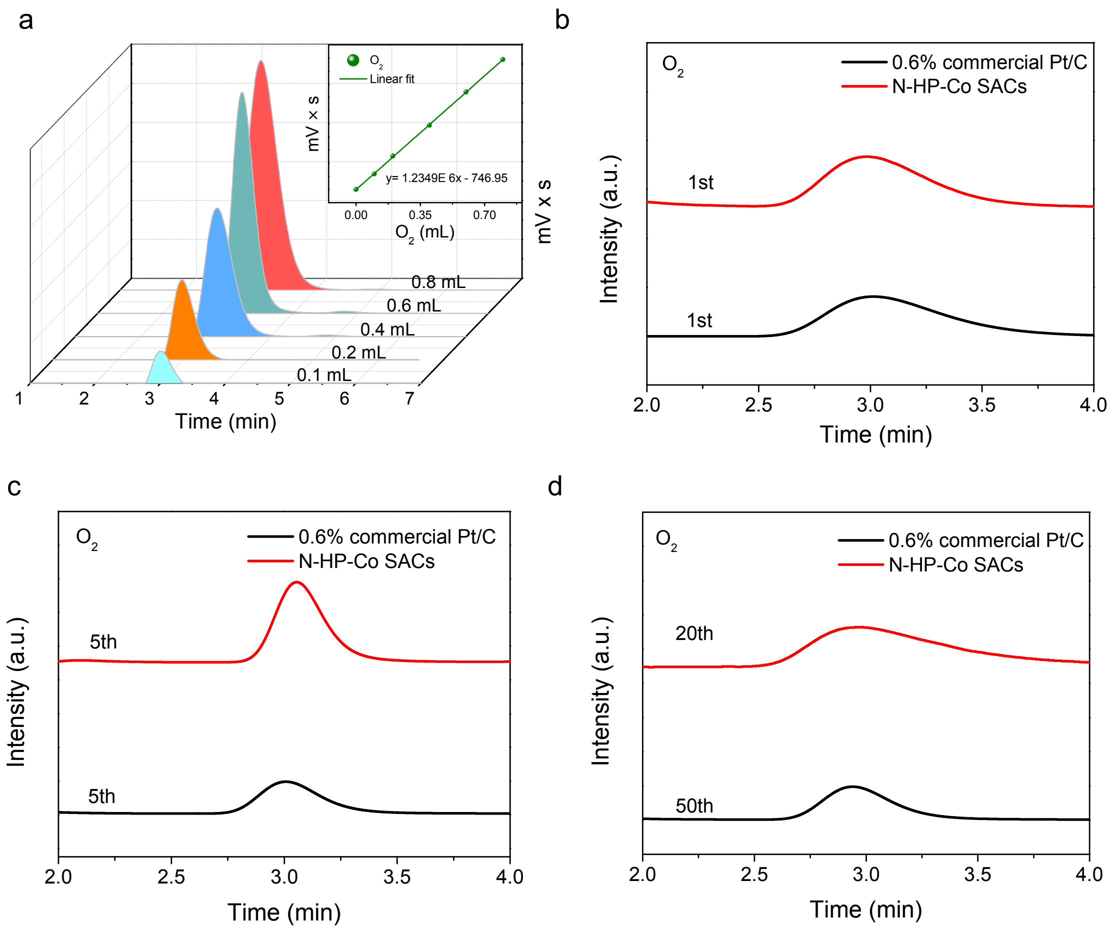

Supplementary Figure 29 | Gas chromatography (GC) signal and a calibration curve of O_2. a. GC signal curves obtained from a thermal conductivity detector (TCD) by injecting different volume O_2 into column. **b**, **c**, **d.** GC signals of the released O_2 after the 1st, 5th and 20th, 50th charging with 0.6% commercial Pt/C cathode (dark) and the N-HP-Co SACs cathode (red) with a thermal conductivity detector (TCD).

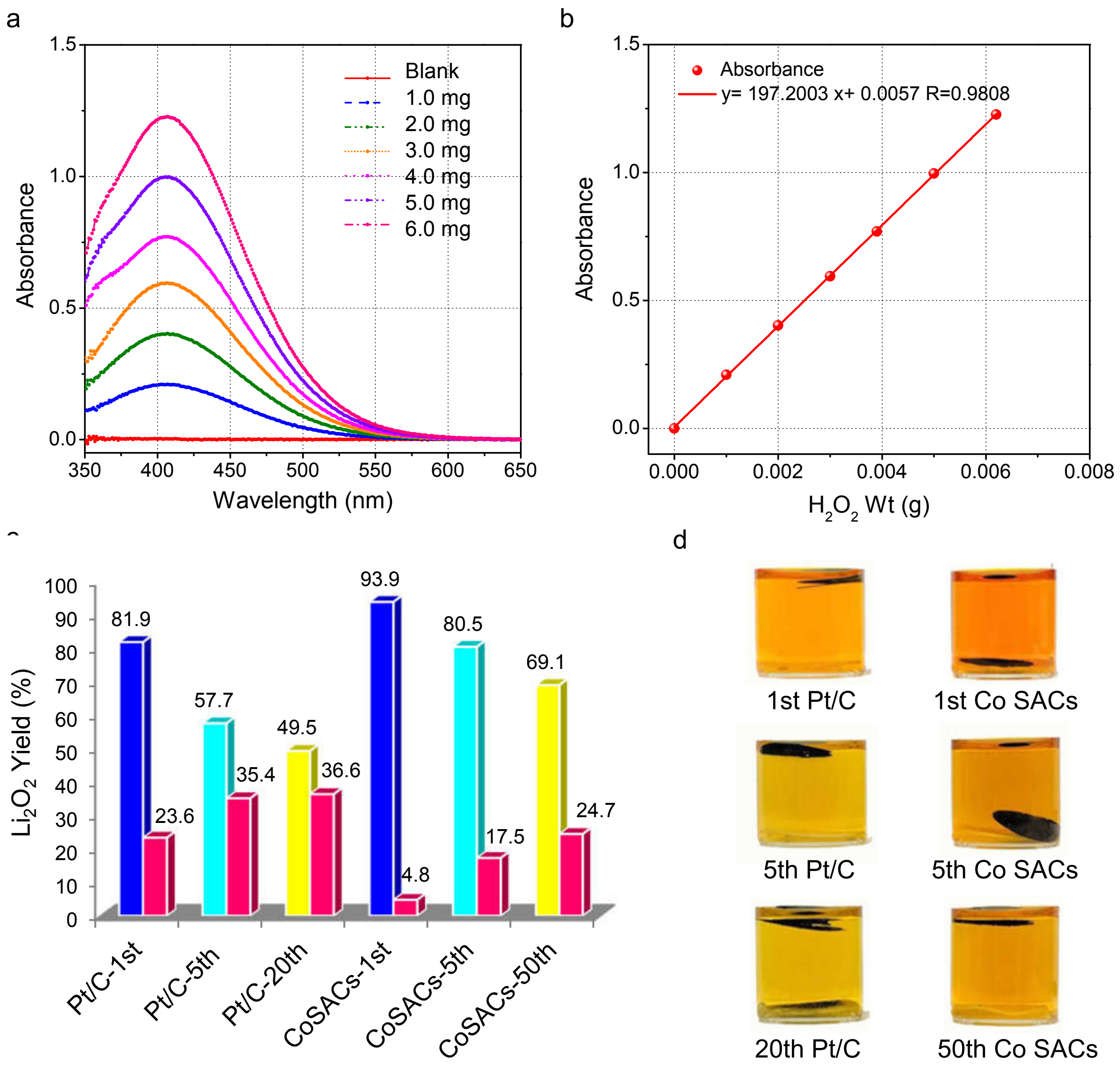

Supplementary Figure 30 | Titration of Li_2O_2. a. UV-vis spectra obtained from the pristine $TiOSO_4$-based standard solution and the solution with the addition of a certain amount of H_2O_2 solution. **b.** Based on the peak located at 405 nm, the related calibration curve towards the hydroperoxide amount can be obtained. In this case, this calibration curve and obtained absorption coefficient (@405 nm) could be employed to quantitatively analyze the amount of Li_2O_2 product. **c.** Li_2O_2 yields obtained from $TiOSO_4$-based UV−vis spectroscopic titration performed on discharged/charged cathodes, wherein the yield is defined as the percentage of obtained Li_2O_2 quantity to the expected calculated quantity based on the ORR capacity (1.0 mAh). **d.** The photographs of titration solutions after immersed with related discharged cathode plates.

Supplementary Tables

Supplementary Table 1 | Co contents analysis by ICP for N-HP-Co SACs catalysts.

Run details	Results
Run	Co (wt%)
1	0.4167
2	0.3825
3	0.5600

Supplementary Table 2 | Structural parameters obtained from the Co K-edge EXAFS fitting.

**Sample**	**Shell**	***CN***	***R*(Å)**	**σ^2**	**ΔE_0**	**R factor**
Co-foil	Co-Co	12*	2.49±0.00	0.0064±0.0003	7.7±0.4	0.0012
CoN_4	Co-N	4.2±0.4	1.91±0.01	0.0089±0.0015	4.1±1.4	0.0025

a. Co-edge (S_0^2=0.732).

b. S_0^2 was set, according to the experimental EXAFS fit of Co foil reference by fixing CN as the known crystallographic value.

c. CN: coordination numbers; R: bond distance; σ^2: Debye-Waller factors; ΔE_0: the inner potential correction. R factor: goodness of fit.

Supplementary Table 3 | The amount of the released gas.

**Sample**	**Cycle numbers**	**mV×s (O_2)**	**V(O_2) (μL)**	**V(O_2)$_{total}$ (mL)**	**Charge efficiency (%)**
0.6% commercial Pt/C	1st	687	1.16	0.36	82.9
	5th	424	0.95	0.28	67.7
	20th	79	0.67	0.20	47.8
N-HP-Co SACs	1st	764	1.22	0.37	87.4
	5th	716	1.19	0.36	84.6
	50th	168	0.74	0.22	52.9

a. These results are obtained on the basis of the GC signal spectra and calibration curves (Supplementary Fig. 29). The current density is 200 mA g^{-1} and the discharge-charge capacity is 1 mAh.

Supplementary Table 4 | Comparison of OER performance for N-HP-Co SACs with other non-precious metal electrocatalysts based on the carbon cathodes in lithium-oxygen batteries.

Catalysts	Specific capacity (mAh g^{-1})	Conversion efficiency (%)	The first Charge Potential (V)	Refs.
MnOx spheres	1000	80	3.5	1
$Li_{0.5}MnO_2$	1000	76	3.5	2
Co-CoO/N-CNR	1000	73.6	3.9	3
CoO mesoporous spheres	1000	72.2	~3.6	4
$Co(OH)_2$@CNS	1000	72.4	3.8	5
NCO@N-rGO ($NiCo_2O_4$)	1000	80.8	3.44	6
Ni-MOF	600	80	~3.7	7
LFO-900	500	76.4	~3.6	8
ZnO/VACNTs	1000	75	3.55	9
MoO_3-xNS_{s-2}	1000	82.6	3.45	10
α-MnO_2	1000	67.5	~4	11
Co_3O_4	1000	65	~4	12
NiO	1000	61.2	~4.25	12
Fe_2O_3	500	61	~4.1	13
$CuCo_2O_4$	1000	74.3	3.7	14
Co-salen/MCNTs@MnO_2	500	82.4	3.4	15
Co_3O_4 nanosheets	500	74.3	3.5	16
CoN_4	1000	80	3.5	17
**N-HP-CoSACs**	**1000**	**86.2**	**3.25**	**This work**

Supplementary Notes

Supplementary Note 1: The SiO_2 can serve as a template for the hollow carbon sphere; we adopt dopamine monomers as the carbon source, because of its rich groups that can easily link with SiO_2.

Supplementary Note 4: Supplementary Fig. 4 shows the Raman spectra of the N-HP-Co NPs and Co SACs samples. Both samples exhibit a D-band at around 1350 cm^{-1} and a G band at around 1585 cm^{-1}, which are characteristic bands of carbonaceous materials. However, because of the content of the Co NPs, we failed to detect the peak of the Co NPs. In detail, the ratio of the G and D bands (I_D/I_G) for Co SACs sample is 0.91 slightly higher than the Co NPs (0.85), indicating the much increased amount of defect sites after Co SACs loaded on the carbon support.

Supplementary Note 5: As shown in Supplementary Fig. 5, the carbon support of the Co SACs was mainly composed of Sp^2 C, which can improve the electronic conductivity.

Supplementary Note 8: In detail, compared with the commonly used catalysts in Li-O_2 batteries, the N-HP-Co SACs electrodes show a higher electrocatalytic activity, indicating that higher surface area and electrical conductivity of the hollow carbon spheres are beneficial to enhance the rate of reaction related to O_2 mass diffusion and exhibit more sites for the Li_2O_2 deposition. Additionally, the tafel plot confirms a superior ORR kinetics of N-HP-Co SACs by a smaller tafel slope of 253 mV $decade^{-1}$ than that of 0.6% commercial Pt/C (323 mV $decade^{-1}$). The power density is calculated to further confirm the excellent activity of N-HP-Co SACs in Supplementary Fig. 8d. The maximum power density of the N-HP-Co SACs (22.0 W g^{-1}) appears at 2.2 V, which is 83.3% higher than that of the 0.6% commercial Pt/C (12.0 Wg^{-1}) at 2.2 V, also

indicating the faster ORR kinetics of N-HP-Co SACs in the Li-O_2 cell. All these improvements provide the strong evidence that not only CoN_4 but also the hollow carbon spheres can enhance the activity towards ORR, confirming the importance of single atoms in the hollow carbon spheres to enhance the catalytic capability in the aprotic Li-O_2 cell.

Supplementary Note 9: To investigate the catalytic activity of the N-HP-Co SACs, the N-HPCSs, N-HP-Co NPs and other catalysts commonly used in Li-O_2 batteries were used for comparison through the full range tests of the first charge-discharge platform, respectively. Unexpectedly, the N-HP-Co SACs exhibited both the ORR and OER performances. In order to further explore the catalytic effect of the Co SACs, we also use 5% commercial Pt/C catalysts for comparison. Remarkably, the Co SACs possesses superior advantages in the discharge capacity and overpotential. The superior catalytic activity is ascribed to the high selectivity towards the Li-O_2 reactions originated from the homogeneity of single-atom sites.

Supplementary Note 11: Supplementary Fig. 11 reveals the first discharge products were mainly Li_2O_2 on the two kinds of cathodes. However, it appeared some amount of the Li_2CO_3 mainly attributing to the carbon passivation with 0.6% Pt/C cathode.

Supplementary Note 16: To further verify the content of the LiO_2 with the N-HP-Co SACs, as depicted in Supplementary Fig. 16, the "*g*" factor of the KO_2 + DMSO solution is around 2.004, which is corresponding to the signal of superoxide[18], and the spectra of the N-HP-Co SACs-containing electrolyte after a half recharge at a current density of 100 mAh g^{-1} displays a similar "*g*" factor ($g = 2.004$). However, compared with N-HP-Co SACs, very weak signal of superoxide can be observed in the electrolytes after the same charging process with 1,000 times magnified intensity for the 0.6% commercial Pt/C, which might be due to the less LiO_2 species obtained in

LiTFSI/TEGDME electrolyte for 0.6% commercial Pt/C catalyst. Therefore, the EPR signal of N-HP-Co SACs after recharge can be assigned to the relatively more LiO_2 species, which is also consistent with the DFT calculations.

Supplementary Note 18: In order to further exclude the effect of the N-doped carbon on the performance of the Li-O_2 batteries, we further considered the graphitic N defective sites as the modeled structures of nitrogen doped graphene. We find that the adsorption energies of the LiO_2 on the different types of N-doped graphene are −2.00, −0.26, −2.37 and −1.55 eV, respectively. In particular, the adsorption energy in **c** is extremely weak because of the physical adsorption when the content of the nitrogen is low, so we speculate that the structure which maybe seldom exist or play a pale pole in our systems. This is consistent with the experimental observation and this result is consistency with the previous reported, further demonstrating the accuracy of our adopted calculation method.

Supplementary Note 21: Thus, for an intrinsic comparison of the OER catalytic activities between the 0.6% commercial Pt/C and N-HP-Co SACs catalysts were conducted under the same condition using a commercial Li_2O_2. We then prepared the electrodes containing catalysts mechanically mixed with the bulk Li_2O_2. The particle-type Li_2O_2 was successfully loaded on the CP as evidenced by the FTIR, PXRD and SEM. Supplementary Fig. 21 presents the charge curves for the cells containing the two different cathodes; one composed of Li_2O_2/the 0.6% commercial Pt/C with super P, another containing Li_2O_2/N-HP-Co SACs, and both of them showed the same behaviour. As detail, the N-HP-Co SACs showed about 500 mV lower charge potential than the commercial Pt/C catalyst at a current density of 200 mA g^{-1}, indicating the high catalytic activity of the Co SACs for the oxidation of commercial Li_2O_2. To confirm the decomposition of Li_2O_2, the cathodes after charge were further investigated by the

PXRD and SEM. For the recharged electrodes with the two kinds of catalysts, the PXRD patterns (Supplementary Fig. 20) demonstrated that the pristine Li_2O_2 was completely disappeared. However, the SEM (Supplementary Fig. 19) related to the Li_2O_2 can still be found on the electrode surface of the 0.6% commercial Pt/C catalysts, which is attributed to the residual Li_2O_2 covering the Pt/C electrode surface. This proves that the N-HP-Co SACs can support the effective oxidation of the Li_2O_2 during charging process, whereas the catalytic activity of 0.6% commercial Pt/C catalyst seems to be inactive to completely decompose Li_2O_2.

Supplementary Note 27: In detail, The FTIR spectra (Supplementary Fig. 26a and b) indicate that the Li_2O_2 on both cathodes are disappeared after the first discharge due to the low charge potential at the first cycle. However, the increasing side products were on the surface of the cathodes with the Pt/C catalyst after 5 and 20th cycles. In contrast, significantly weaker peaks are ascribed to the byproducts on the cathode with the N-HP-Co SACs after subsequent cycles. In addition, ^{1}H NMR spectroscopy on the cathode with Co SACs (Supplementary Fig. 27b) is much less than that on the cathode with Pt/C catalysts (Supplementary Fig. 27a) during cycling, which agrees with FTIR results. These observations demonstrate that our Co SACs possess much more stable battery chemistries in the Li-O_2 batteries. According to the above research studies, we can point out that albeit the noble-based catalysts such as Pt/C catalysts have relatively high active towards the decomposition of Li_2O_2, they can also accelerate the side reaction in an ether-based electrolyte system, that side reactions are proven to primarily result from the intrinsic instability of the electrolyte, carbon, and binder against the nucleophilic attack by O^{2-} ions. The information of byproducts from parasitic reactions could be further interpreted by the instability of the Li-O_2 battery systems followed by comparing spectroscopic results between commercial Pt/C and N-HP-Co SACs electrodes.

Supplementary Methods

Chemicals and Materials: Cobalt (II) acetylacetonate, Dopamine hydrochloride, and 5% commercial Pt/C, and Tris-buffer were purchased from Aladdin Reagent, KO_2, Li_2CO_3, CH_3CO_2Li and HCO_2Li were purchased from Sigma-Aldrich. Li_2O_2 was purchased from Acros. CP (carbon paper) was purchased from Torray. Ethanol, N-methyl-2-pyrrolidinone, TEGDME, LiTFSI, Nitrotetrazolium blue chloride dye (2, 2-bis (4-Nitrophenyl)-5, 5-diphenyl-3, 3-(3, 3-dimethoxy-4, 4-diphenylene) ditetrazolium chloride and deuterium oxide were purchased from Aladdin Reagent.

Synthesis of the N-HP-Co NPs catalyst: The N-HP-CoNPs catalyst was prepared through a similar process except for usage of cobalt acetylacetonate (100 mg).

Synthesis of the N-HP-Co-free catalyst: The N-HP-Co-free catalyst was synthesized by the the same method without adding any cobalt acetylacetonate.

Synthesis of the 0.6% commercial Pt/C catalyst: The 5% commercial Pt/C catalysts and Super P were mixed in Dimethyl Formamide (DMF) with a weight ratio of 1:3.

Electrolytes: Lithium bis(trifluoromethane sulfonyl)imide (LiTFSI) was the preferred lithium salt for all experiments reported in this manuscript. 1 M LiTFSI in tetraethylene glycol dimethylether (TEGDME) was prepared. The water content in electrolyte is measured by Karl Fischer titration.

XAFS Analysis and Results: The acquired EXAFS data were processed according to the standard procedures using the ATHENA module implemented in the IFEFFIT software 3 packages. The EXAFS spectra were observed by deduction of the post-edge background from the overall absorption and the normalization with respect to the edge-jump step. Subsequently, the $\chi(k)$ data were Fourier transformed to real (R) space using a hanging windows ($dk = 1.0$ Å^{-1}) to separate the EXAFS contributions from different

coordination shells. To obtain the quantitative structural parameters around central atoms, the least-squares curve parameter fitting was carried out using the ARTEMIS module of IFEFFIT software packages. The following EXAFS equation was applied:

$$\chi(k) = \sum_j \frac{N_j S_0^2 F_j(k)}{kR_j^2} \cdot exp[-2k^2\sigma_j^2] \cdot exp\left[\frac{-2R_j}{\lambda(k)}\right] \cdot sin[2kR_j + \phi_j(k)] \quad (1)$$

S_o^2 is the amplitude reduction factor, $F_j(k)$ is the effective curved-wave backscattering amplitude, N_j is the number of neighbors in the jth atomic shell, R_j is the distance between the X-ray absorbing central atom and the atoms in the jth atomic shell (backscatter), λ is the mean free path in Å, $\phi_j(k)$ is the phase shift (including the phase shift for each shell and the total central atom phase shift), σ_j is Debye-Waller parameter of the jth atomic shell (variation of distances around the average R_j). The functions of $F_j(k)$, λ and $\phi_j(k)$ were calculated with the ab initio code FEFF8.2[19].

Rotating disk electrode (RDE) voltammetry measurement: Glassy carbon disk electrodes (0.25 mm OD) coated with 30 μL 1 mg mL^{-1} catalyst and Super P suspension made by mixing with 0.5 wt% lithiated Nafion in isopropanol were used as a working electrode, and Li metal was used as a counter and reference electrode. 1 M LiTFSI in tetra (ethylene) glycol dimethyl ether (TEGDME) is used as the organic electrolyte. The Li-O_2 cells were fabricated in an Ar-filled glove box (H_2O < 0.1 ppm, O_2 < 0.1 ppm). The CV curves were recorded from 3.2 to 2.2 V at a scan rate of 5 mV s^{-1} with O_2-saturated electrolyte under electrode rotation speed of 900 rpm.

Superoxide detection using nitrotetrazolium blue chloride: Nitrotetrazolium blue chloride dye was applied to selectively detect superoxide species and differentiate it from peroxide. Anhydrous DMSO was used to disperse the dye. Cells containing Co

SACs and commercial Pt/C carbon cathodes were prepared and recharged to the same capacity at different stages. Dye/DMSO solution was immediately injected into the coin cell negative case after cell disassembly. The separator with commercial KO_2 was taken out for photography and comparison. The procedure was carried out in the Ar-filled glovebox.

Electron paramagnetic resonance (EPR): The Li-O_2 batteries with 0.6% Pt/C and N-HP-Co SACs cathodes during recharging were disassembled in a glovebox filled with Ar atmosphere, and electrolyte of the recharged battery was transferred to a sealed nuclear magnetic tube before exposed to air. After being taken out from the glovebox, the nuclear magnetic tube was immediately transferred to the Dewar bottle filled with liquid nitrogen. Further, the EPR spectra (−196 °C) were recorded on a Bruker E500 EPR spectrometer at 9.4 GHz with a modulation frequency of 100 kHz and a modulation amplitude of 2 G in a quartz finger Dewar filled with liquid nitrogen.

In situ differential electrochemical mass spectrometry (DEMS) analysis and the Gas chromatography (GC) signals: The in situ DEMS was performed using an OmniStar GSD 320 system (Pfeiffer Vacuum) mass spectrometry, wherein argon gas was chosen as the carrier gas. The detection system is based on a commercial quadrupole mass spectrometer, a LAND CT2001A multi-channel battery testing system connected with computer and a DEMS cell. The DEMS Swagelok cell is designed with two capillary tubes as purge gas inlet and outlet. The cell is assembled in an Ar glove box. For the gas chromatography (Techcomp GC-7900) measurements, a glass chamber containing the Li-O_2 battery following discharging was filled with Ar atmosphere. The gas was then collected for analysis after the battery recharged. O_2 was analyzed with a thermal conductivity detector with Ar as the carrier (limit of detection: 10 p.p.m.).

$TiOSO_4$-based Li_2O_2: The Ti^{4+}-based solution was prepared with $TiOSO_4$ (1g), H_2SO_4

(1g, 98%) and ultrapure DI-water (50 mL). Then, the harvested cathodes, the glass fiber (GF) separator soaked with cycled electrolyte solution were directly dropped into 5 mL Ti^{4+}-based solution without washing and evaporating procedures. The solution was vigorously shaken for 30s to promote the complete reaction of Li_2O_2 with H_2O. The involved reaction is as follows:

$$Li_2O_2 + 2H_2O = 2LiOH + H_2O_2 \quad (2)$$

$$(pH < 3)\ Ti^{4+} + H_2O_2 + 2H_2O = H_2TiO_4\ (orange) + 4H^+ \quad (3)$$

$$(pH < 3)\ Ti^{4+} + H_2O_2 = Ti(H_2O_2)^{4+}\ (orange) \quad (4)$$

$$(pH\ 3\sim6)\ Ti^{4+} + H_2O_2 = Ti(HO_2)^{3+}\ (colorless) + H^+ \quad (5)$$

$$(pH\ 3\sim6)\ Ti^{4+} + H_2O_2 = Ti(O_2)^{2+}\ (yellow) + 2H^+ \quad (6)$$

Then, based on the corresponding calibration curve harvested by the certain amount of hydroperoxide solution (Supplementary Fig. 30), UV-vis spectroscopy of the obtained orange (yellow) solution is used to quantitatively analyze the amount of Li_2O_2.

Supplementary References

1. Zhang, S., Wen, Z., Lu, Y., Wu, X. & Yang, J. Highly active mixed-valent MnOx spheres constructed by nanocrystals as efficient catalysts for long-cycle Li-O_2 batteries. *J. Mater. Chem. A* **4**, 17129-17137 (2016).

2. Hu, Y., Zhang, T., Cheng, F., Zhao, Q., Han, X. & Chen, J., Recycling application of Li-MnO_2 batteries as rechargeable lithium-air batteries. *Angew.Chem. Int. Ed.* **54**, 4338-4343 (2015).

3. Hyun, S. & Shanmugam, S. Mesoporous Co-CoO/N-CNR nanostructures as high-performance air cathode for lithium-oxygen batteries. *J. Power Sources* **354**, 48-56 (2017).

4. Shang, C. et al. Compatible interface design of CoO-based Li-O_2 battery cathodes with long-cycling stability. Chen, *Sci. Rep.* **5**, 8335 (2015).

5. Zhu, J., Metzger, M., Antonietti, M. & Fellinger, T. P. Vertically aligned two-dimensional graphene-metal hydroxide hybrid arrays for Li-O_2 batteries. *ACS Appl. Mater. Interfaces* **8**, 26041-26050 (2016).

6. Gong, H. et al. High-loading nickel cobaltate nanoparticles anchored on three-dimensional N-doped graphene as an efficient bifunctional catalyst for lithium-oxygen batteries. *ACS Appl. Mater. Interfaces* **8**, 18060-18068 (2016).

7. Hu, X., Zhu, Z., Cheng, F., Tao, Z. & Chen, J. Micro-nano structured Ni-MOFs as high-performance cathode catalyst for rechargeable Li-O_2 batteries. *Nanoscale* **7**, 11833-11840 (2015).

8. Cong, Y. et al. Cation segregation of a-site deficiency perovskite $La_{0.85}FeO_{3-\delta}$ nanoparticles toward high-performance cathode catalysts for rechargeable Li-O_2 battery. *ACS Appl. Mater. Interfaces* **10**, 25465-25472 (2018).

9. Fan, W., Wang, B., Guo, X., Kong, X. & Liu, J. Nanosize stabilized Li-deficient $Li_{2-x}O_2$ through cathode architecture for high performance Li-O_2 batteries. *Nano Energy* **27**, 577-586 (2016).

10. Zhang, S. et al. Self-catalyzed decomposition of discharge products on the oxygen vacancy sites of MoO_3 nanosheets for low-overpotential Li-O_2 batteries. *Nano Energy* **36**, 186-196 (2017).

11. Débart, A., Paterson, A., Bao, J. & Bruce, P.G. α-MnO_2 nanowires: a catalyst for the O_2 electrode in rechargeable lithium batteries. *Angew. Chem. Int. Ed.* **47**, 4521-4524 (2008).

12. Debart, A., Bao, J., Armstrong, G. & Bruce, P. G. Effect of catalyst on the performance of rechargeable lithium/air batteries. *ECS Trans.* **27**, 225-232 (2007).

13. Li, Z. L. et al. Fe_2O_3 nanoparticle seed catalysts enhance cyclability on deep (dis)charge in Aprotic Li-O_2 batteries. *Adv. Energy Mater.* **8**, 1703513 (2018).

14. Liu, Y. et al. Facile synthesis of spinel $CuCo_2O_4$ nanocrystals as high-performance cathode catalysts for rechargeable Li-air batteries. *Chem. Commun.* **50**, 14635-14638 (2014).

15. Hu, X. et al. MCNTs@MnO_2 nanocomposite cathode integrated with soluble O_2-carrier co-salen in electrolyte for high-performance Li-air batteries. *Nano Lett.* **17**, 2073-2078 (2017).

16. Wang, J. K. et al. Boosting the electrocatalytic activity of Co_3O_4 nanosheets for a Li-O_2 battery through modulating inner oxygen vacancy and exterior Co^{3+}/Co^{2+} ratio. *ACS Catal.* **7**, 6533-6541 (2017).

17. Yoon, K. R. et al. Brush-Like cobalt nitride anchored carbon nanofiber membrane: current collector-catalyst integrated cathode for long cycle Li–O_2 batteries. *ACS Nano* **12**, 128-139 (2018).

18. Lindsay, D. & Garland, D. ESR spectra of matrix-isolated lithium superoxide. *J. Phys. Chem.* **91**, 6158–6161 (1987).

19. Han, A. J. et al. A polymer encapsulation strategy to synthesize porous nitrogen-doped carbon-nanosphere-supported metal isolated-single-atomic-site catalysts. *Adv. Mater.* **30**, 1706508 (2018).
